# A Paradigm Revolution or Just Better Resolution—Will Newly Emerging Superresolution Techniques Identify Chromatin Architecture as a Key Factor in Radiation-Induced DNA Damage and Repair Regulation?

**DOI:** 10.3390/cancers13010018

**Published:** 2020-12-23

**Authors:** Martin Falk, Michael Hausmann

**Affiliations:** 1 Institute of Biophysics, The Czech Academy of Sciences, 612 65 Brno, Czech Republic; 2 Kirchhoff Institute for Physics, Heidelberg University, 69120 Heidelberg, Germany; hausmann@kip.uni-heidelberg.de

**Keywords:** DNA damage and repair, DNA double-strand breaks (DSBs), ionizing radiation, linear energy transfer (LET), chromatin architecture, ionizing radiation-induced foci (IRIFs), DSB repair pathway choice and hierarchy, superresolution microscopy, single-molecule localization microscopy (SMLM)

## Abstract

**Simple Summary:**

Radiation-induced double-strand breaks (DSBs) are the most toxic and most difficult to repair DNA lesions and are very heterogeneous. These characteristics place considerable demands on the selection of the most suitable repair mechanism at each individual damage site. Here, we review the current knowledge on this still enigmatic process and hypothesize that it critically involves the local chromatin architecture at the micro- and nanoscales, later manifested in the architecture of DSB repair foci (i.e., IRIFs).

**Abstract:**

DNA double-strand breaks (DSBs) have been recognized as the most serious lesions in irradiated cells. While several biochemical pathways capable of repairing these lesions have been identified, the mechanisms by which cells select a specific pathway for activation at a given DSB site remain poorly understood. Our knowledge of DSB induction and repair has increased dramatically since the discovery of ionizing radiation-induced foci (IRIFs), initiating the possibility of spatiotemporally monitoring the assembly and disassembly of repair complexes in single cells. IRIF exploration revealed that all post-irradiation processes—DSB formation, repair and misrepair—are strongly dependent on the characteristics of DSB damage and the microarchitecture of the whole affected chromatin domain in addition to the cell status. The microscale features of IRIFs, such as their morphology, mobility, spatiotemporal distribution, and persistence kinetics, have been linked to repair mechanisms. However, the influence of various biochemical and structural factors and their specific combinations on IRIF architecture remains unknown, as does the hierarchy of these factors in the decision-making process for a particular repair mechanism at each individual DSB site. New insights into the relationship between the physical properties of the incident radiation, chromatin architecture, IRIF architecture, and DSB repair mechanisms and repair efficiency are expected from recent developments in optical superresolution microscopy (nanoscopy) techniques that have shifted our ability to analyze chromatin and IRIF architectures towards the nanoscale. In the present review, we discuss this relationship, attempt to correlate still rather isolated nanoscale studies with already better-understood aspects of DSB repair at the microscale, and consider whether newly emerging “correlated multiscale structuromics” can revolutionarily enhance our knowledge in this field.

## 1. Global Versus Local DSB Repair Pathway Selection and Regulation

Double-strand breaks (DSBs) are the most deleterious type of DNA lesion and are induced in DNA by ionizing radiation, radiomimetic chemicals and cellular processes [1,2,3]. Theoretically, a single DSB may lead to cell death or initiate carcinogenesis if left unrepaired or repaired improperly [4]. After exposure to high doses of sparsely ionizing radiation or even low doses of densely ionizing radiation, there is a serious risk that numerous and possibly clustered DSBs will not be repaired in a timely manner, leading to separation of broken DNA ends, misrejoining of these ends, and formation of often lethal chromosomal aberrations. These events probably explain why fast repair mechanisms have evolved and are preferred by organisms with large genomes [5]. However, a fast rate of repair may be at the expense of repair accuracy, resulting in smaller mutations, some of which may be carcinogenic and thus no less dangerous than larger mutations. Hence, damaged cells have to solve a serious repair dilemma and maintain a careful balance between repair *speed* and *fidelity*.

**Figure 1:**
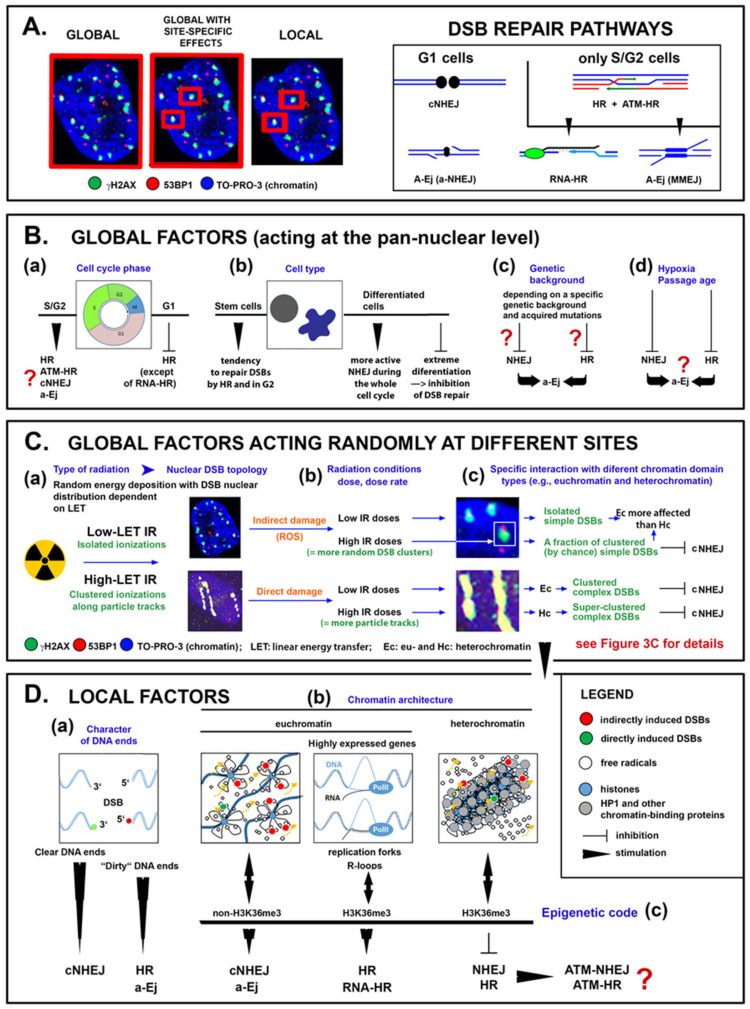
Schematic representation of prominent pan-nuclear-acting (global) factors, global factors acting randomly at different sites, and site-specific (local) factors that participate in the selection of DSB repair pathways at individual DSB damage sites. (**A**) Left: definition of the nuclear competence of repair pathway-selecting factor types; the area of competence is indicated by the red frames. Right: DSB repair pathways plus their principles and mutual transitions depending on the cell cycle phase (G1 vs. S/G2 cells). (**B**) Examples of global factors (a–d) having a pancellular effect on DSB repair pathways and their selection. Repair pathways preferred or affected by each of these factors and the character of their influence are suggested. (**C**) The relationship between three interdependent factors related to irradiation that have a global mode of action but locally specific effects—radiation LET, irradiation conditions (dose, dose rate) and chromatin architecture (a–c)—is proposed, together with the potential outcomes of these factors on DSB repair pathway selection. (**D**) Diversity of radiation-induced DSB damage sites in terms of (a) the characteristics of broken DNA ends, the architecture and function of damaged chromatin (b), and the epigenetic code. The influence of these local factors on DSB repair pathways is indicated. For interactions between factors B, C and D and their joint effect on the activation of particular DSB repair pathways, see Figures 3 and 4.

In mammals, the two main repair pathways with these opposite repair strategies are the fast but error-prone nonhomologous end joining (NHEJ) and the much slower but usually precise homologous recombination (HR) [6]. Unsurprisingly, NHEJ and HR utilize, in principle, different repair mechanisms (Figure 1A) specialized to cope with different repair targets and scenarios. In addition, alternative repair pathways (hereafter and in the figures collectively referred to as alternative end joining; A-Ej) have been identified (Figure 1A), which extend or back up the conventional repair pathways in situations that remain incompletely understood [7,8,9,10,11,12]. These pathways combine aspects of both NHEJ and HR mechanisms to various degrees [13], as reflected in their problematic and still inconsistent categorization. Most often reported are alternative NHEJ (aNHEJ; also known as backup NHEJ, bNHEJ), single-strand annealing (SSA), and microhomology-mediated end joining (MMEJ), which differ in the requirement for some repair proteins, extent of DNA end resection, and length of homology needed for recombination [7,14]. NHEJ and HR always offer—because of their opposite advantages and disadvantages—only a compromise solution, indicating the requirement for precise regulation of mutual repair pathway competition and cooperation within the repair network.

The cell cycle has long been accepted as the main DSB repair pathway-regulating factor since the demand for sister chromatids (i.e., repair templates) restricts HR to S/G2 phase (Figure 1A,B). Although HR can utilize homologous chromosomes or repetitive sequences as alternative templates in some organisms (including mice [15]) or under specific circumstances [16], in mammals, the resection of DNA ends (necessary to initiate HR) is generally actively inhibited in G1 cells to prevent recombination via these potentially mutagenic mechanisms [17,18,19,20]. Hence, NHEJ has historically been considered the dominant repair pathway in human cells, while broken DNA end resection is considered the critical factor for determining the pathway of repair and signaling [21]. However, notably, embryonic stem cells are essentially dependent on HR [22,23,24], and HR templated by homologous chromosomes is the predominant repair pathway throughout the cell cycle in these cells in mice [25]. The activity of NHEJ and HR thus evidently depends on additional factors uniformly affecting the cell nucleus (herein referred to as “global factors”) (Figure 1B). In addition to the cell cycle and cell type, as already mentioned [23,24], the degree of cell differentiation [26], corresponding chromatin condensation [27], genetic background [28,29,30] and physiological status, such as age [31] and hypoxia [32], are the most frequently reported determining factors. For instance, cell differentiation accompanied by chromatin condensation generally suppresses HR in favor of NHEJ but may even completely prevent DSB repair in extreme cases [27]. The effects of genetic background on DSB repair can be seen especially in cancer cells or even precancerous syndromes, where abundant genome aberrations frequently shift the repair pathway balance in either direction relative to that in wild-type cells [28,29,30]. Tumors also often exhibit varying degrees of hypoxia, which preferentially downregulates HR or even generally reduces the repair capacity of cells [32], as does cell aging [31].

Of note, Carreau et al. [33] reported that the physoxic range in human tissues is between 11% and 1%, challenging the results of in vitro experiments on DSB repair, which are usually uniformly performed in ~20% O_2_.

**Figure 2:**
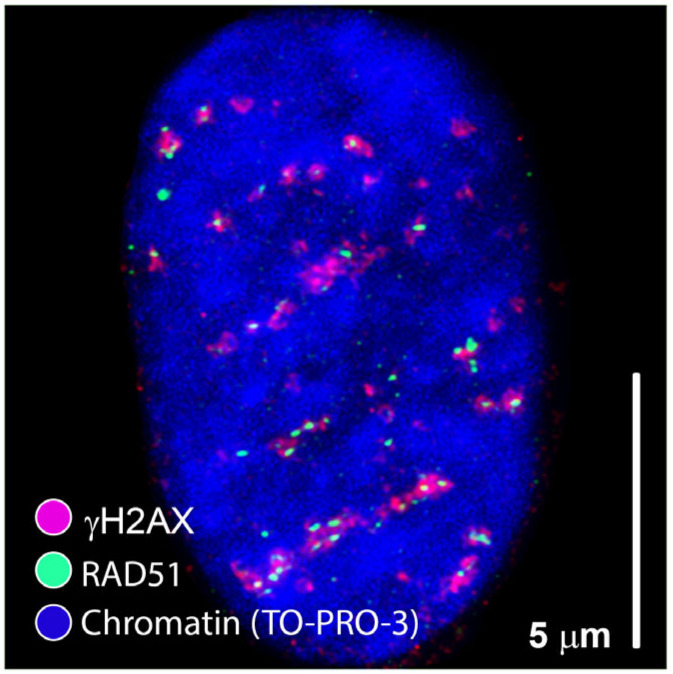
HR in a representative cell exposed to high-LET radiation. A G2-phase nucleus in a normal human skin fibroblast irradiated with ^15^N ions (182.9 keV/μm, 13.0 MeV/n, 10° angle between the ion beam and the cell monolayer) and fixed 4 h post irradiation is displayed with immunofluorescently labeled γH2AX (red) and RAD51 (green) repair foci (i.e., IRIFs). As demonstrated by the figure and extensive literature, HR seems to be a preferred repair pathway in G2 cells exposed to high-LET radiation. However, not all DSBs in this single nucleus are being repaired by this (preferred) pathway (note the γH2AX sites that are not colocalized with RAD51), suggesting that cells must consider numerous globally and locally acting factors (see Figure 1) when selecting a particular pathway at individual damage sites. In other words, each damage site may be repaired via a different mechanism even under the influence of factors that confer a global preference for one particular pathway. A maximum image composed of superimposed individual optical confocal slices (0.05 μm thick) acquired with a Leica SP5 microscopy system (Leica) is shown after deconvolution using Lightning (Leica) software. Chromatin-dense (“heterochromatin”, stained intensely blue) and chromatin-sparse (“euchromatin”, weakly stained) domains are visualized with blue fluorescence (TO-PRO-3).

Interestingly, NHEJ rejoins ~70% of DSBs induced by sparse ionizing radiation in both G1 and G2 cells [34,35,36,37]. In G1 cells, greater NHEJ involvement could be expected if NHEJ is the only canonical pathway active at this stage of the cell cycle. In G2 cells, on the other hand, the corresponding proportion is surprisingly high, considering that error-free HR is available. This finding thus implies some important aspects of DSB repair regulation regarding the role of the cell cycle. First, nonstandard HR activity (e.g., RNA- or homologous chromosome-templated HR) occurs in G1 cells [16,25,38], possibly supported by alternative repair pathways [11]. Second, repair pathways compete for individual DSB targets within single nuclei in all cells [39] (reviewed in [38]). Figure 2 shows the nucleus of a human dermal fibroblast irradiated with accelerated ^15^N ions, which form complex DSBs that generally require HR repair. Not all DSB lesions are repaired by this mechanism, as evidenced by the absence of γH2AX foci colocalization with the RAD51 protein (even assuming that colocalization may occur at some DSBs later). The competition of repair pathways for DSB targets and the existence of alternative repair pathways collectively indicate the importance of local factors (the complex environment) at each damage site in the regulation or even the selection of the most suitable repair mechanism.

Among these factors, the type of incident radiation and the irradiation conditions should be discussed first. Because of its unique mode of action, (ir)radiation can be considered a global factor with a locally specific effect (Figure 1C) [23]. The topology of DSBs in the cell nucleus, which crucially impacts the mechanism and efficiency of repair, is defined by the physical characteristics (especially the linear energy transfer; LET) of the radiation and irradiation conditions. As shown in Figure 1C, exposure to low-LET radiation causes isolated DSBs to be distributed throughout the nucleus, while multiple DSBs formed by dense ionizing (high-LET) radiation are closely concentrated along the particle trajectory. However, for both radiation types, the severity and character of DNA lesions at individual damage sites, i.e., their multiplicity [40] and complexity (combination with other non-DSB damage types) [41], differ dramatically, because radiation releases its energy randomly along the photon or particle path [42,43].

**Figure 3:**
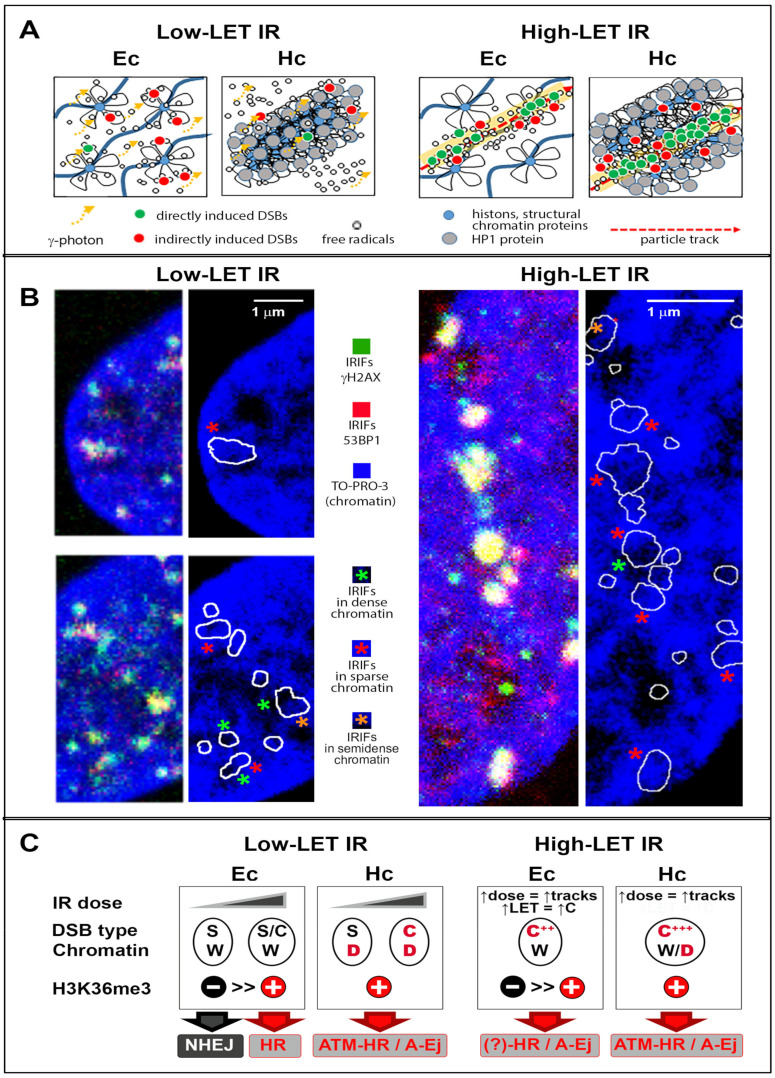
The relationship between the physical properties of the incident radiation, chromatin architecture, character of DSB damage and repair mechanism. (**A**) Euchromatin (Ec) and heterochromatin (Hc) interact differently with ionizing radiation. This interaction is also dependent on the physical parameters of the incident radiation, especially its linear energy transfer (LET). After low-LET irradiation, highly compacted heterochromatin is better protected than euchromatin against indirect radiation DNA damage due to its lower hydration, higher occupation by chromatin-binding proteins, and, in turn, lower accessibility of DNA to harmful free radicals (e.g., [76]). However, heterochromatin is the more critical target for high-LET particles that (mostly) damage DNA directly. In contrast to the indirect damage mediated by radicals, the damage generated by concentrated energy release from high-LET particles cannot be prevented by the condensed, protein-rich heterochromatin architecture. As heterochromatin offers more DNA targets per unit than euchromatin, its interaction with energetic particles leads to the formation of more complex DSBs than in euchromatin, as described in [103,104] and shown in panel B. (**B**) Confocal micrographs (0.3 μm-thick slices) showing IRIF foci (γH2AX—green, 53BP1—red) and their distribution relative to one another and relative to chromatin architecture in normal human skin fibroblasts irradiated with low-LET (left panel) and high-LET radiation (right panel). The right columns in both panels show only IRIF borders (white line) superimposed over chromatin stained with TO-PRO-3 to reveal its density and architecture at IRIF sites (blue to black gradient: high- to low-density chromatin). Larger IRIFs representing highly complex DSBs are marked with green, orange or red asterisks depending on their location in sparse, semicondensed or condensed chromatin, respectively. IRIFs generated at the boundary between dense and decondensed chromatin or occupying both types of chromatin domains are indicated by two asterisks of corresponding colors. (**C**) The proposed mutual interplay of the physical properties of the incident radiation, radiation dose, and local chromatin environment with respect to the character of generated DSBs and activated repair mechanisms. Specifically, the radiation LET, radiation dose, character of the generated DSBs (S–simple, C–complex, C***–extremely complex DSBs), architecture of the affected chromatin domains (W–weakly stained open, low-density (eu)chromatin domains; D–dense and condensed (hetero)chromatin domains), and presence of the H3K36me3 epigenetic mark (characteristic of highly expressed loci and heterochromatin) are considered. In cells exposed to high-LET radiation (right panels), increasing the radiation dose increases the average number of particles hitting the nucleus, while increasing LET increases the complexity of the generated DSBs. The factor(s) having the major (or dominant) role in repair pathway selection are displayed in red. In the case of heterochromatin domains irradiated with high-LET radiation (the rightmost image), the character of chromatin architecture is indicated as W/D; this means that chromatin within originally chromatin-dense (D) domains may be seriously fragmented by transpassing particles, which leads to different degrees of or even complete disintegration, mimicking an open chromatin architecture (W). For heterochromatin exposed to low-LET radiation, two preferred repair pathways have been described (indicated as ATM-HR/A-Ej). In cells exposed to high-LET radiation, HR is generally preferred; if HR is repressed in G1 cells, A-Ej pathways appear to be used instead (indicated as HR/A-Ej).

Moreover, chromatin is hierarchically organized at multiple scale levels, which leads to the formation of structurally and functionally distinct chromatin domains [44,45,46,47], with which radiation interacts in a specific way and creates DNA lesions with different requirements for repair (reviewed in [48,49,50,51,52]). The chemical properties of the broken DNA ends were the first local factor (Figure 1D) recognized to dramatically affect the ability of repair enzymes to rejoin DSBs [53]. However, it soon became clear that DSB repair is controlled not only (bio)chemically but also physically. Among physical local factors, DSB complexity [21,40] and several interconnected characteristics of damaged chromatin—its epigenetic code [54], transcription activity [54], 3D architecture [53,55,56,57] (Figure 3A,B), and nuclear position [58,59]—appear to play critical though still disputed roles [53] (Figure 1D). Site-specific combinations of local factors thus introduce a new level of complexity into the regulation of DSB repair, suggesting that the assembly of repair complexes and functioning of repair proteins depend on the nontrivial local environment at individual DSB sites (Figure 3C). In addition to the microscopic studies discussed in the following chapters, other modern approaches, such as CRISPR-Cas9 technology, have provided evidence confirming the crucial relevance of local factors in repair pathway selection. For example, Brinkman et al. [60] demonstrated that cell irradiation prior to subsequent Cas9 cleavage does not change the characteristic repair mechanism at Cas9-DSB sites, i.e., MMEJ.

Considering these observations collectively, it can be predicted that global factors (Figure 1B) restrict the spectrum of available or preferred repair pathways, while globally acting factors with site-specific effects (Figure 1C) and local factors (Figure 1D) ultimately determine the selective pathway activation at each individual damage site. For instance, exposure to densely ionizing radiation [61,62], high or very low doses of radiation [63,64], and fractionated irradiation [65] have been recognized to increase the proportion of DSBs, whose repair is dependent on HR (Figure 1C). In contrast, as mentioned above, some global factors inhibit or at least suppress this repair pathway (Figure 1B) [66,67]. Thus, the preferences for a particular repair pathway conferred by individual factors may be contradictory and lead to the selection of different repair pathways at each DSB lesion (Figure 2). Accordingly, HR-related DNA damage response proteins may be activated at damage sites that are ready for template-free end-to-end repair [8,9,10,39]. The time window for considering the above-outlined multiple factors before an irreversible selection step is made may thus be relatively wide [21,39]. Computer simulations to reproduce the DSB repair kinetics, which correlate better with entwined repair pathways (at least in the initial phase) than with a strictly competitive scenario [39], support this possibility. Thus, questions arise as to whether and how repair complexes (i.e., ionizing radiation-induced foci; IRIFs) differ at individual DSB sites depending on the repair pathway and/or chromatin architecture of the damaged domain. It is also necessary to identify the repair phase during which differences in IRIF architecture can initially be observed and to determine whether the architecture of early IRIFs can select the subsequent repair mechanism.

**Figure 4:**
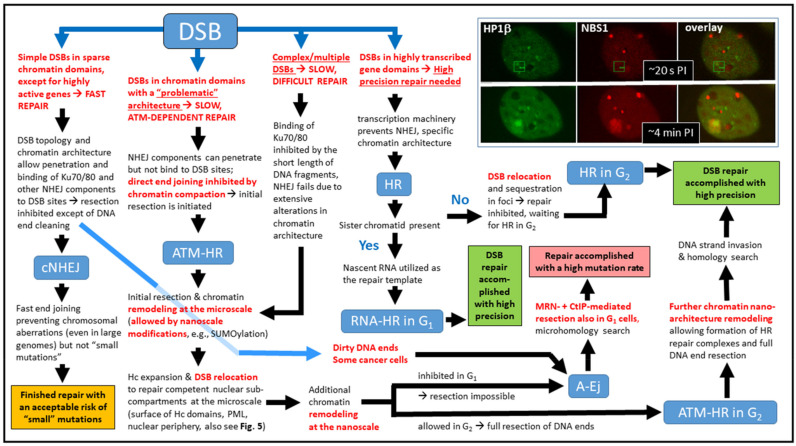
Schematic summary of the main steps of a possible decision-making scenario for particular repair pathway activation at individual DSB sites. The influence of the DSB properties and architecture of the affected chromatin domain is highlighted. Insert: DSB damage is accompanied by architectural reorganization (decondensation) of a heterochromatin domain at the microscale, which is necessary for repair continuation in this chromatin domain type (see also Figure 5). Shown is also infiltration of the affected domain by NBS1 protein, confirming DSB induction by a UV laser (see Falk et al. 2014 [77]). HP1β was labeled in living MCF7 cells by GFP- and NBS1 by RFP tagging.

The questions outlined are addressed in the following chapters. From different points of view and at different (micro- to nano-) scale levels, we discuss how chromatin architecture, a critical but still unexplored local decision-making factor, influences the formation of IRIFs and participates in repair pathway selection. Without claiming to be complete, Figure 4 summarizes the main steps in the proposed scenario, together with consequences of the decision sequences on repair reliability. Importantly, throughout the article, we understand the term “architecture” in a complex sense—it refers to the molecular composition of chromatin and IRIFs as well as to the spatial relationships (topology) between individual molecules and, at higher organization levels, their clusters (foci). For chromatin, the term is approximately equivalent to the term “higher-order structure”.

## 2. Is Regulation of DSB Repair Physically Controlled Through Chromatin and IRIF Architecture?

The revolution in our understanding of DSB repair pathways and their regulation in the context of the cell nucleus was initiated by the discovery of so-called IRIFs (Figure 3B) [68]. At DSB sites, histone H2AX becomes phosphorylated on serine 139 (γH2AX) immediately after irradiation, and this modification eventually spreads over a 2-megabase region of the adjacent chromatin [69]. γH2AX foci then, in cooperation with other epigenetic modifications present or inserted at DSB sites, participate in DNA damage signaling and serve as docking platforms for the assembly of repair complexes [70,71,72]. Because of their large size and abundance of recruited proteins, these complexes can be microscopically visualized as IRIFs (see [73,74] for the methodology). Microscale analyses of spatiotemporal IRIF composition and behavior post irradiation performed by our team [40,75,76,77] and many other teams (reviewed, e.g., in [48,49,50,72,78,79,80]) have provided extensive insights into DSB repair mechanisms and their regulation and efficiency at individual damage sites in the physiological context of the natural chromatin environment. Unsurprisingly, IRIF analysis has become a powerful tool in DNA damage and repair research (e.g., [73,81]), as demonstrated, for instance, by the more than 100 records published in 2019—2020 found by searching the PubMed database [82] (https://pubmed.ncbi.nlm.nih.gov) with the keywords “repair focus + DSB”.

**Figure 5:**
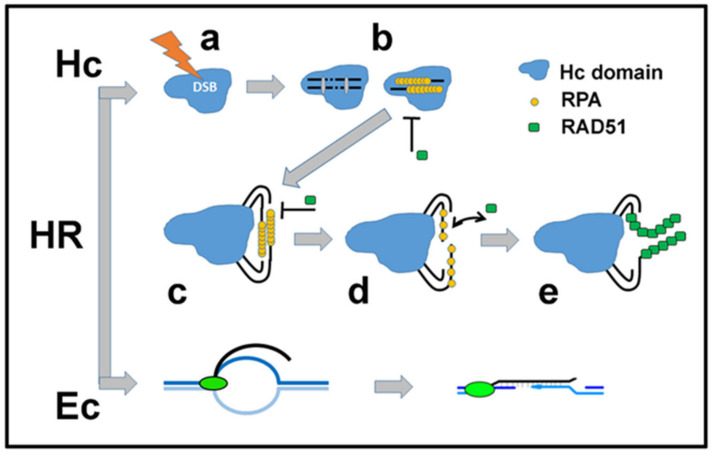
The proposed mechanism underlying the multistep regulation of homologous recombination (HR) based on chromatin architecture at the multiscale. HR is activated by the microarchitecture of heterochromatin (Hc) domains and highly transcribed gene loci (euchromatin; Ec). In euchromatin, the HR machinery preferentially recognizes transcription-related structures (transcription forks, R-loops) within intensively transcribed loci and ensures their precise repair (bottom); however, in heterochromatin, HR is the pathway of choice for the complex (dense) architecture but may be very risky due to the presence of repetitive sequences. Hence, HR in heterochromatin must be precisely regulated in multiple steps associated with or even controlled by changes in chromatin architecture. (**a**) Double-strand break (DSB) induction within the heterochromatin domain is followed by (**b**) resection of DNA ends, which is initiated and proceeds within the Hc domain. Single-stranded DNA (ssDNA) chains are protected by RPA proteins, but their interaction with the RAD51 recombinase is prevented by the domain architecture. (**c**) Subsequently, the heterochromatin domain decondenses, and the damaged chromatin protrudes out of the domain, allowing its additional interactions with repair proteins. However, the binding of RAD51 to ssDNA remains inhibited until chromatin remodeling occurs at the nanoscale (**d**). After this remodeling, RPA is replaced by RAD51 (**e**), which allows homology search and recombination. Eventually, homology search is supported by global (pan-nuclear) chromatin decondensation [107] and reorganization [114]. At actively transcribed gene loci, HR can proceed either by the classical mechanism (in the G2 phase of the cell cycle) or by using nascent RNA as the repair template (in G1 phase), as illustrated in the figure.

IRIF formation and disassembly have been studied in great detail for opened, genetically active euchromatin and condensed, genetically inactive heterochromatin (Figure 3) (reviewed in [48,50,51,52,83,84,85,86,87]). In our previous study [76], we used IRIF microscopy to compare radiation damage and repair also for regions of increased gene expression (RIDGEs) [88,89] and anti-RIDGEs, representing domains even more structurally and functionally distinct than euchromatin and heterochromatin. Numerous studies have revealed the core proteins of the NHEJ pathway to be sufficient for successful end joining in euchromatin, while the same process in heterochromatin requires ATM and additional NHEJ proteins [36]. This auxiliary NHEJ machinery is likely essential for decondensation of damaged heterochromatin domains and/or DNA end resection, which control the continuation of repair [90,91,92,93,94]. The need for decondensation is somewhat surprising here, as heterochromatin does not seem to be inaccessible to repair proteins [95,96], and its decondensation may interfere with the effort to spatially stabilize the free DNA ends. This paradox implies that heterochromatin architecture poses a barrier to repair ([77], reviewed in [91]) (Figure 5).

Indeed, this implication was functionally confirmed by the observation that ATM-defective cells do not exhibit NHEJ deficiency in heterochromatin provided that KAP-1 or HP1 expression is inhibited concomitant with ATM mutation [55,97]. On the other hand, disruption of heterochromatin architecture by HP1γ depletion with siRNA substantially increased micronucleus formation, showing that heterochromatin architecture dramatically influences not only DNA transcription and replication [98] but also DNA repair [97].

Regarding both the G1 and G2 phases of the cell cycle, however, the principles of repair pathway selection driven by chromatin architecture have usually been discussed only for G2 cells, where the two major pathways—NHEJ and HR—compete for DSB targets. In G2 cells, the complex heterochromatin architecture steers repair towards HR. Interestingly, although DNA resection and coating of single-stranded DNA (ssDNA) with RPA have already occurred within heterochromatin domains, the broken DNA ends must protrude out of the condensed chromatin before RPA proteins can be replaced by the RAD51 recombinase and recombination can eventually occur (Figure 5) [55,99,100] (reviewed in [101]). Notably, other DSB types repaired by HR also show similar behavior. Ribosomal genes must protrude from nucleoli to be repaired, and in some organisms, problematic damaged loci are transported and anchored to the nuclear envelope (reviewed in [101,102]). HR thus appears to be strictly regulated by chromatin architecture at several hierarchical levels of scale and function.

These experimental findings are confirmed by a theoretical biophysical model that describes densely packed heterochromatin as an entropic spring [105]. The entropic freedom of a DSB region enhances its mobility, an advantage for homologous repair, and helps to relocate broken DNA ends to the periphery of the heterochromatin domain. This spring-like relaxation improves the access of repair proteins to DSB and allows HR regulation. The formation of a break naturally triggers a reaction of the whole system driven by thermodynamic rules of entropy maximization, resulting in movement of the loose ends of broken chromatin outside of tight domains. Considering that the repair machinery reacts to all kinds of DSBs, it becomes evident that passive processes governed by entropy contribute significantly to chromatin architecture reorganization. The results of these processes affecting damage site accessibility could thus be predicted to play a key role in the subsequent DSB repair processes. Physically controlled processes thus seem to offer the possibility of simple and universal regulation of some important steps in DSB repair pathways. Consistent with this assumption, ATM, which is required for the repair of heterochromatin in both the G1 and G2 phases of the cell cycle, does not seem to be involved in DSB escape from the heterochromatin domain [55] but rather to participate in the modification of damaged chromatin nanoarchitecture after relocation (discussed later). Alternatively, the physical forces and (bio)chemical mechanisms may work closely together to more precisely regulate heterochromatin decondensation (Figure 5). In any case, physical factors may be predicted to regulate DSB repair pathway performance, with most steps occurring in combination with (bio)chemical factors but some steps even occurring independently.

The nature of cooperation/competition between all regulatory factors is thus dictated by the physical forces, energy and information contained in physical structures (chromatin domains); biochemical interactions; and the overall specific environment at the particular damage site. Consistent with this idea (i.e., indicating the importance of the structure per se), it should be emphasized that the damaged domains undergo relaxation, although the original epigenetic marks of heterochromatin—H3K9me3 and H4K20me3—remain unchanged during repair [97,106]. The two interdependent regulatory systems of chromatin—its physical architecture and the epigenetic code codefining this architecture—are thus temporarily uncoupled [94]. Hence, the chromatin architecture itself may control repair at certain stages, while the epigenetic code retains the information to restore the original domain architecture after rejoining the DSB [107] (Figure 5).

Highly expressed genes must be repaired precisely and, recover their function as quickly as possible. Moreover, the DSB repair machinery in intensively transcribed regions may initially collide with the transcription machinery. Highly expressed genes thus represent difficult targets for repair and a type of chromatin that specifically activates HR in G2 phase [54,108] (Figure 5). For genes highly transcribed in G1 phase, previous studies noted clustering of IRIFs, which was associated with NHEJ inhibition and waiting for HR in G2 phase [109]. This strategy also ensures the preservation of important genetic information if DNA breaks arise during a cell cycle phase when HR is not available. The scenario might be supported by the knowledge that the G1/S checkpoint is not fully activated for hours post irradiation and that the G2/M checkpoint responds only to an amount of damage in excess of 10 to 20 DSBs [110]. Interestingly, the G1/S checkpoint could be even less stringent than the G2/M checkpoint, according to the study of Chao et al. [111], who monitored the activation of these checkpoints in real time.

However, more recent data suggest that active genes may also employ HR in G1 phase by utilizing nascent RNA as a template for precise repair (Figure 5) (reviewed in [38]). As DNA end resection is inhibited in G1 cells, an alternative model with classical nonhomologous end joining (cNHEJ) taking advantage of the same principle (RNA-templated repair) has also been proposed [112]. Despite this possibility, it can be predicted that the blockade of DNA end resection (otherwise conditioning HR) is circumvented by ongoing transcription, also generating ssDNA strands. However, the exact mechanism by which HR is initiated remains to be explained. As the functionally opposite and epigenetically distinct domains of highly transcribed genes and silent heterochromatin share one common epigenetic mark, H3K36me3, this mark may recruit the HR machinery [54]. The architecture of DNA:RNA hybrids and the architecture of the active (opened) chromatin domains on the whole may also play an important role because inhibition of transcription has been shown to prevent HR in G1 phase [38,54,113], probably also because of RNA template loss.

Accumulating evidence suggests that although both active genes and heterochromatin are repaired by HR (Figure 3C, Figure 4), these structurally and functionally opposite domains require different chromatin remodeling and regulatory steps [115] (Figure 5). The architecture of heterochromatin hinders both NHEJ and HR [54], with the latter mechanism unlocked only after relocation of DSBs out of dense heterochromatin regions and subsequent additional chromatin remodeling, as already described (reviewed in [101]). Hence, this multistep architectural reorganization (Figure 5) ensures spatiotemporal orchestration of the HR pathway in the context of chromatin. This strategy first prevents illegitimate recombination between repetitive sequences within the heterochromatin domain [99] and, subsequently, fine-tunes recombination activity so that HR is efficient but hyperrecombination does not occur [116]. In the domains of active genes, on the other hand, collapsed transcription forks must be resolved, and transcription of damaged loci must be silenced to prevent the collision of the transcription machinery with the repair machinery [117]. In addition, distinct RNAs begin to be transcribed from damaged sequences of active genes, which probably facilitates HR [38,118]. Hence, IRIFs forming at DSB sites within these structurally and functionally opposite (heterochromatin vs. active gene) chromatin domains could be predicted to have mutually different architectures. The dynamic architecture of nascent IRIFs, in turn, may participate in the regulation of repair, as discussed in the following chapter.

Heterochromatin remodeling leading to decondensation was also observed in G1 cells [75], i.e., in association with NHEJ. Because the priority of NHEJ is to stabilize the free DNA ends as quickly as possible, this phenomenon is quite paradoxical. We can thus hypothesize that decondensation of heterochromatin domains is necessary for the assembly of both the HR and NHEJ repair complexes. Alternatively, in G1 cells, activation of A-Ej pathways accompanied by DNA end resection and recombination between microhomologies (MMEJ) may explain this phenomenon. Collectively, these results suggest that the local architecture of chromatin can affect the choice of repair pathway [7,54,86,119] and is a key determinant of the pathway mechanism and efficiency [91,100].

The functional significance of the architecture of both chromatin domains and IRIF foci in DSB repair pathway selection and/or progression can also be strongly supported by our preliminary observation that γH2AX foci induced by high-LET radiation undergo different micromorphological developments during NHEJ and HR; a similar phenomenon was observed also for 53BP1 foci in different cell types (discussed later) [120]. Moreover, γH2AX foci in irradiated cells start to colocalize with RAD51, a late-HR actor, at 30 min post irradiation, suggesting that HR can be initiated very early post irradiation, i.e., in parallel to NHEJ. Since nonsynchronized cells were studied, one explanation for this phenomenon may be the faster appearance of HR-associated resection in S-phase cells than in G2-phase cells [121]. However, supporting the role of chromatin architecture in repair pathway selection and regulation, disruption of damaged chromatin domains by high-LET radiation can facilitate the access of DSB repair proteins to lesions commonly accessible only after decondensation of damaged domains, thus accelerating HR initiation [122,123]. Together, these findings might challenge the model, which states that HR can be activated only after unsuccessful NHEJ [55], provided that NHEJ failure does not occur very soon after irradiation. A current study [124] indicates that the activation of NHEJ vs. HR may not be directly dependent on resection. Therefore, the architecture of both DSB lesions and damaged domains may steer repair in a certain direction from the outset. However, this scenario is still insufficiently explored, with some studies emphasizing only the role of DNA end resection [21,36,125]. Remarkably, at least one of those studies also implied that chromatin nanoarchitecture at the damage site may play an important role in regulating resection [125].

In addition, binding of repair proteins to DSB sites was observed to depend on the localization of the DSB within the cell nucleus [58]. We can thus speculate that the local effects of chromatin architecture, its remodeling during repair, and the specific location of a DSB in the cell nucleus influence the nanoarchitecture of IRIFs and, in turn, the repair mechanism. However, whether the architecture of nascent IRIFs also participates in selecting the repair mechanism initiated at a particular DSB site remains unknown. Despite supportive indications in this direction outlined above, some single-locus studies in mice and Drosophila, for instance, did not reveal substantial changes in the balance between NHEJ and HR after shifting of the affected sequence between heterochromatin and euchromatin states [115,126].

The collective results of IRIF exploration at the microscale show that chromatin architecture plays an irreplaceable role in DSB repair regulation at individual damage sites. However, to better understand the relationship between chromatin architecture, IRIF architecture, and repair mechanisms, we must reveal how chromatin and individual repair proteins interact at the molecular level, i.e., at the nanoscale. The first results on this topic are introduced in the next chapter.

## 3. First Insights into DSB Repair and its Regulation at the Nanoscale

As described above, heterochromatin is a typical example of a repair-repressive chromatin architecture that requires relaxation to allow repair, regardless of the repair pathway. Moreover, heterochromatin reorganization is one of mechanisms regulating HR. Experiments with inhibition of ATM and other relevant proteins demonstrated that microscopic rearrangement of the damaged domains must be followed by chromatin remodeling at the nanoscale to allow RAD51 binding and IRIF formation [101,116], i.e., the critical step for DNA strand displacement and recombination. Standard confocal microscopy has thus suggested that the nanoarchitecture of chromatin and IRIFs could play a crucial and functional role in DSB repair, although this method cannot provide direct insight into these processes. Thus, even before the era of superresolution light microscopy, it could have been predicted that microarchitectural chromatin domain reorganization opens up space for additional adjustments at the nanoscale. This possibility suggests multistep (sequential) spatiotemporal regulation of HR, with the sequence of these steps determined by the hierarchical chromatin/IRIF architecture and mediated by its reorganization at multiple scale levels.

From our discourse about chromatin repair, we recapitulate all the factors that impact repair pathway decisions: radiation type, dose and LET; genetic activity at the damaged site; the local chromatin environment at the damaged site and the location of the damage site relative to the nucleus; the number and density of damage events; the cell cycle state; etc. All these factors have to be considered at each damage site [83], while DNA end stabilization and initial repair pathway decisions have to be made within seconds. Thus, it may be surprising that with only very few exceptions, repair processes function perfectly during the whole life of a cell and at each base pair throughout the genome. Considering this exceptional robustness and reliability of repair, as well as the number of decision-making factors, it is reasonable to hypothesize that there is a mechanism that integrates these factors into a composite signal that is more easily evaluable by cells than each parameter alone. In the previous chapter, we presented the evidence suggesting that this mechanism/signal could be based on physical laws and can automatically control repair pathway selection through the recruitment of the appropriate repair protein machinery to a damage site and the establishment of the repair (chromatin/IRIF) environment.

Especially during the initial phase of repair, many proteins that play either multiple or redundant roles in different repair pathways or even appear not to be necessarily needed accumulate at DSB sites. For example, ATM is required only for repair in heterochromatin; however, it is recruited by both heterochromatin and euchromatin damage. This situation complicates research on the role of IRIFs in repair pathway selection and regulation but also supports the idea that other factors in addition to chromatin domain-specific epigenetic codes are relevant to recruiting particular key repair proteins. If so, the local chromatin architecture could selectively interact with incoming repair proteins [127], organize their spatiotemporal recruitment to DSB sites [128,129], and assist the assembly of initial repair complexes. Indeed, IRIF formation has been shown to be regulated both temporally and spatially in the context of the surrounding chromatin [130,131,132].

Hence, we can assume that the architecture of initial IRIFs not only reflects the local chromatin architecture at DSB damage sites but also contributes to the regulation of additional protein binding to IRIFs and to the stability of particular repair complexes—and thus to IRIF maturation and site-specific selection of the most suitable repair pathway at individual DSB sites. Heterochromatin, for example, relaxes after damage induction; thus, it does not prevent the entry of repair proteins but may interact with given proteins differently than does euchromatin [77,133]. The condensed fractal architecture of heterochromatin may thus regulate how—rather than if—DSB repair proteins are recruited to IRIFs. Macromolecular crowding in condensed heterochromatin blocks the access of other molecules to a large domain volume, slows the diffusion of macromolecules, and shifts the binding reactions of these molecules to the bound state [95]. These effects may differ for individual repair proteins [77].

Microscopic research of DSB repair is further complicated by the fact that some proteins of which only a few molecules are needed, do not form microscopically distinguishable IRIFs and can thus be visualized only by superresolution methods. Electron microscopy has yielded surprising results regarding the focal accumulation of repair proteins in euchromatin and heterochromatin. Lorat et al. [52,103], who analyzed the nuclear distribution of various repair proteins in cells irradiated with low-LET and high-LET radiation showed that at two time periods post irradiation (0.5 and 5 h), γH2AX, MDC1, and 53BP1 can be detected only in heterochromatin domains positive for H3K9me3, while the Ku70/80 heterodimer can be detected in both euchromatin and heterochromatin. This observation strongly suggests the involvement of the micro- and/or nanoarchitecture of chromatin and, subsequently, IRIFs in the selection and/or propagation of a particular repair pathway. However, the absence of the indicated protein foci in euchromatin has not been confirmed by any other technique and contradicts the results of confocal microscopy.

Methodologically, this contradiction may be explained by a gap in resolution and thus a gap in knowledge in the 50 nm to 200 nm scale range. Whereas the strength of electron microscopy lies in the low-10-nm scale range, optical confocal microscopy covers resolutions above 200 nm. Thus, for decades during the second half of the 20th century, the scale range between electron and confocal microscopy, although highly relevant for biomolecular dynamics, seemed to be obscured for gaining scientific insights. Therefore, great hopes are currently placed in emerging studies using superresolution light microscopic techniques [134,135], which cover this critical gap of visualization and approach a resolution of 10 nm while preserving the advantages of optical microscopy.

**Figure 6:**
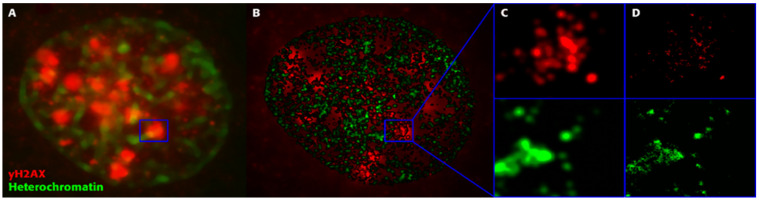
Superresolution imaging of a breast cancer (SkBr3) cell during DSB repair after exposure to 1 Gy X-rays. From such images, characteristic molecular arrangements during repair are elucidated. (**A**) Overview image acquired by widefield microscopy. The blue square (2 µm × 2 µm) encloses a typical γH2AX focus. (**B**) Superresolution SMLM image of heterochromatin (green) and γH2AX foci (red), with the γH2AX overview image in the background indicating the reduced z-slice depth in the SMLM image reconstructed from the label point coordinates. (**C**) Magnification of the marked region (2 µm × 2 µm) in the SMLM image with Gaussian blur but without the background image; the two color channels are separated in the upper and lower images. (**D**) The same image as (**C**) but with maximum precision of label points (each point corresponds to a single fluorescent molecule of the indicated antibody). Maximum precision means the highest image resolution that can be obtained from the SMLM data set. (Note: In all images, the blue squares enclose an area of 2 µm × 2 µm and can be used as scale bars.).

We recently introduced single-molecule localization microscopy (SMLM) [136,137] to simultaneously analyze the architecture of damaged chromatin domains and IRIFs at the nanoscale (see, for example, Figure 6; compare the widefield image, panel A, with SMLM images, panels B–D) [106,138,139]. SMLM is one of the superresolution (nanoscopy) techniques established in recent decades [140]. In addition to having an improved resolution of approximately 10 nm, SMLM is renowned for providing quantitative data on 3D localization (coordinates) and other signal parameters of individual molecules of interest without a need for complicated image analysis. Several SMLM and other nanoscale studies have shown that IRIFs have an internal nanoarchitecture, with nanoclusters of γH2AX and individual proteins occupying nonoverlapping space [141,142].

**Figure 7:**
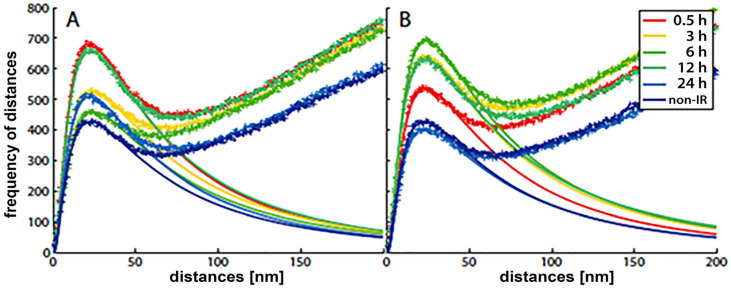
Frequency histograms of pairwise distances of H3K9me3 heterochromatin label points in breast cancer cell (SkBr3) nuclei at different times post irradiation with two doses of X-rays: (**A**) 500 mGy, (**B**) 4 Gy. The distributions of the crosses represent the experimentally measured results. The smooth curves, which follow a logarithmic Gaussian distribution, are fitted curves of the peaks below 100 nm, indicating cluster formation in heterochromatin. According to Ripley’s interpretation, the linearly increasing experimental curves describe a random behavior of molecule positions, i.e., the dense clusters are embedded in an environment of randomly, less densely arranged H3K9me3 marks. Non-IR: the nonirradiated control.

With the SMLM data matrix of molecule coordinates, Ripley’s metrics for pairwise distance frequency histograms [139,143] can be applied to evaluate structures, molecular clusters, or spatial distributions of label points and their dynamic rearrangements during repair (Figure 7; compare panels A and B for 500 mGy and 4 Gy exposure to X rays) [106,141,144]. Using these approaches of structure elucidation from distance frequency patterns, together with newly developed mathematical topological tools based on persistent homology [145], we showed (for SkBr3 cells exposed to 1 Gy X-rays) that the topological similarity and, thus, the nanoarchitecture of γH2AX clusters depends on the distance of the clusters from heterochromatin (Figure 6) [106]. High topological similarities were also found for 53BP1 clusters in repair foci along high-LET ^15^N particle tracks in human dermal fibroblasts and the U87 glioblastoma cell line [120]. More generally, this finding means that the architecture of γH2AX and 53BP1 clusters is not random and depends on the chromatin environment at DSB sites, consistent with the results of high-resolution ChIP-seq mapping of γH2AX spreading from multiple DSBs induced at annotated positions in human DIvA cells [146]. This finding shows that phosphorylation follows a highly stereotyped pattern governed by the original (predamage) chromatin architecture. Provided that the chromatin architecture dictates γH2AX spreading, it is reasonable to suppose that the architecture of nascent γH2AX foci subsequently affects downstream repair events. Such events could be the binding and organization of repair proteins (such as MDC1 and 53BP1) to IRIFs, the insertion of epigenetic marks (e.g., ubiquitin) into IRIFs, and, in turn, the determination of the architecture of maturating or already dissolving IRIFs [147].

Indeed, the roles of numerous proteins in DSB repair dramatically depend on the specific conditions. The 53BP1 protein generally inhibits resection and promotes NHEJ [148]. However, at some DSB substrates, it shows the opposite effects. For instance, it stimulates resection and switches NHEJ to MMEJ [8,11,149]. In addition, 53BP1 enhances repair fidelity independent of the repair pathway [150]. Hence, 53BP1 and some other proteins, such as BRCA1, probably establish structural platforms that support the recruitment and assembly of the repair machinery in specific ways, dictated by integrated information from multiple global and local factors (reviewed in [151]). Strikingly, 53BP1 and RIF1 were only recently discovered to form an autonomous functional module that stabilizes three-dimensional chromatin topology at sites of DNA breakage [150].

Our SMLM analysis also revealed that the nanoarchitecture of γH2AX foci in heterochromatin shows a higher mutual similarity than γH2AX foci in euchromatin (unpublished data). These greater differences between IRIFs in euchromatin probably reflect the variability in the expression intensity across euchromatin loci, in contrast to the rather uniformly silenced heterochromatin. On the other hand, heterochromatin experiences especially extensive architectural reorganization associated with repair initiation and progression. Thus, γH2AX foci in euchromatin may still reflect the variable original architectures of differently expressed genomic domains, but the architecture of γH2AX foci in heterochromatin has already adopted the features of remodeling. Our results thus suggest that remodeling processes at different sites in heterochromatin broadly follow the same principles, indicating that the same repair mechanism is active across these sites. This situation is in contrast to the variable repair of DSBs in structurally and functionally heterogeneous euchromatin.

In addition, using SMLM, we showed that the formation kinetics and architecture of 53BP1 foci differ for normal (nontransformed) and tumor cells, represented in the study by human dermal fibroblasts and highly radioresistant U87 glioblastoma cells, respectively [120]. The data currently being processed seem to suggest that γH2AX, RAD51, and potentially other repair proteins also form IRIFs with cell type-specific kinetics and architecture. These differences might contribute to differences between the cells in repair pathway utilization and capacity.

Other breakthrough studies supporting the idea that IRIF nanoarchitecture reflects the repair mechanism or even significantly contributes to the repair pathway choice were published by Reindl et al. [121,152]. By using STimulated Emission Depletion (STED) microscopy [153], another well-established superresolution fluorescence microscopy technique, the authors showed that two nanoscale subzones exist within HR- but not NHEJ-associated IRIFs. Specifically, the resection zone and the zone of surrounding modified chromatin were recognized in [121,152] and in our preliminary unpublished analyses. Furthermore, IRIFs formed by different repair proteins that have specific functions in the NHEJ and HR pathways, such as 53BP1, BRCA1 and RAD51, were clearly shown to have different architectures ([121,152] and our unpublished results). Finally, mutual reorganization of 53BP1, BRCA1 and RAD51 proteins in the frame of IRIFs correlated with switching between NHEJ and HR [121,152]. Hence, the HR, NHEJ, and perhaps A-Ej pathways seem to form IRIFs with characteristic architectures.

Several other studies on IRIF nanoarchitecture have recently been published but cannot be discussed here due to space limitations. However, these studies focus on IRIF ultrastructure rather than the relationship of this ultrastructure to the selection of repair pathways [154]. Future experiments on synchronized cells, cells with altered/manipulated DSB repair pathways, or cells exposed to high-LET ions, as discussed below, are expected to provide more accurate insights into the relationship between the repair mechanisms and nanoarchitecture of particular chromatin domain types and IRIFs.

## 4. Specificities for High-LET Particle Radiation

Cells exposed to high-LET particles (Figure 3) contain a matrix of all possible combinations of global and local damage-to-repair scenarios at individual DSB sites that can occur after irradiation (Figure 1, and Figure 3C). This characteristic offers a unique opportunity to study the activation of different repair pathways in the context of chromatin architecture. High-LET particles generate predominantly complex DSB lesions with a minority of single DSBs [40]. In addition, the architecture of domains directly traversed by the particle is extensively disrupted, while domains damaged outside the track by delta electrons are better preserved. Microdosimetric measurements have thoroughly shown that the mutual topology of DSBs is another important factor that determines the repair mechanism [40,141,152] and its efficiency (Figure 4). The repair process of complex DSBs is not yet clear; however, a unique pathway specific for this type of damage has not been identified.

In the G2 phase of the cell cycle, complex DSBs are removed preferentially by HR because the Ku70/80 heterodimer can bind to short chromatin fragments generated by high-LET radiation only with difficulty. Complex DSBs thus suppress cNHEJ [155,156] and favor recombination regardless of the damaged chromatin domain type [157] (Figure 2 and Figure 3C). This strategy should reduce the risk of short fragment misalignments or deletions. In G1 cells, however, HR can occur only within transcriptionally active sequences. In addition, Ku-dependent cNHEJ is generally inhibited in cells exposed to high-LET radiation, as already mentioned. Repair of high-LET damage in G1-phase cells is thus likely shifted towards alternative repair mechanisms (i.e., A-Ej) that, like HR, involve some extent of DNA end resection, but with recombination based on repeat- or microhomology-mediated pairing [158,159,160]. Indeed, MMEJ—associated with CtIP and MRN activity—has been shown to be activated by radiation-induced “dirty” DNA ends that are refractory to cNHEJ [8,149]. MMEJ is thus suspected by some authors to repair most DSBs in irradiated cells [8,149,158]. Importantly, the fraction of high-LET radiation-induced DSBs removed by MMEJ is significantly higher than that of low-LET radiation-induced DSBs [161], which suggests that cNHEJ suppresses MMEJ and that high-LET radiation inhibits cNHEJ. Considering the different topologies of DSBs/IRIFs generated by low-LET and high-LET radiation, this change in repair pathway employment supports the hypothesis that architectural aspects are involved in repair pathway selection at individual DSB sites.

DSB repair in cells damaged by high-LET particles is problematic even if HR is used. Although preferred, HR may easily become deregulated. High-LET particles may significantly disrupt the architecture of (hetero)chromatin domains (Figure 2 and Figure 3), possibly inflicting serious consequences, especially for repair in heterochromatin, where repeats are frequent and architectural damage eliminates its protective barrier against illegitimate recombination (see Section 2). Accordingly, Jakob et al. identified small γH2AX foci within heterochromatin soon after irradiation of cells with high-LET radiation [96], while this phenomenon was not observed in studies using low-LET radiation [55].

Furthermore, HR at multiple DSB lesions produced by high-LET radiation may be dangerous even if the architecture of the affected domain remains preserved and damaged chromatin successfully relocates out of heterochromatin. As already explained in Section 2, the chromatin decondensation necessary for repair in heterochromatin also has a dark side, i.e., it increases the mobility of DSBs and thus the risk of chromosomal translocations. This shortcoming of repair in heterochromatin is particularly important in cells damaged by high-LET particles. Due to the nuclear topology of DSBs, i.e., their accumulation along the particle path, the movement of numerous DSBs from heterochromatin may easily cause mutual clustering of these DSBs, DNA end misrejoining, and the formation of complex chromosomal translocations/aberrations.

In addition to differences between high-LET radiation and low-LET radiation in direct effects on repair processes, high-LET radiation interacts differently than low-LET radiation with euchromatin and heterochromatin, showing that chromatin architecture determines the susceptibility of functionally distinct chromatin domains to radiation damage (Figure 3) [76,97]. Finally, it should be emphasized that the architecture of chromatin and the physical parameters of the incident radiation do not influence DNA damage and repair separately but in mutually interact (Figure 3C). High-LET radiation generates more DSBs in heterochromatin than in euchromatin [162], although heterochromatin protects DNA better against damage from low-LET radiation than does euchromatin (Figure 3A) [48,76]. The combination of the high density of ionization events and DNA targets in heterochromatin may allow the formation of more complex DSBs (Figure 2 and Figure 3) [50]. Hence, although some studies failed to observe any difference between the complexity of DSBs in heterochromatin and euchromatin [97,163], it has become increasingly apparent that the influence of chromatin architecture on DSB repair is complex and multilevel. The consequences of DSB misrepair are discussed in detail for specific conditions (type of radiation, chromatin domain, and repair) in the next chapter and summarized in Table 1.

## 5. Incorrect DSB Repair, Formation of Chromosomal Aberrations and Cancer

Repair pathways dramatically differ in their reliability. HR repairs DSBs slowly but generally with high precision [6,164]. In contrast, alternative pathways are highly mutagenic in principle, as they promote deletions between involved repeats or short homologous sequences and, because of their slow kinetics, allow the formation of translocations [8,10,13,165]. Fast cNHEJ reduces DSB roaming in the nucleus and thus the risk of chromosomal aberrations, but short deletions or insertions are generated at DSBs induced by radiation because of the need to “clean” (short resection) chemically incompatible free DNA ends. NHEJ thus poses a relatively small risk to human health, as approximately 98% of the human genome does not contain genes.

Previously, however, the accuracy of repair pathways has been shown to depend not only on the repair mechanism itself but also on its interaction with specific chromatin substrates [44,166,167,168,169]. Remarkably, HR can also be risky under certain circumstances, e.g., when it occurs in heterochromatin that is DNA-dense and rich in repetitive sequences [99,170,171]. If HR is accidentally initiated too early within the heterochromatin domain, before the broken DNA ends are spatially separated from repeat-rich loci, repair may be completed with an illegitimate recombination between the single-stranded DNA ends and one of the repeat blocks [170]. Subsequently, heterochromatin dysfunction induced by aberrant HR with repeat blocks may cause, for instance, chromosome segregation errors, transposon activation, or replication stress, which may provoke genetic malfunction and carcinogenic transformation [172,173]. HR in heterochromatin thus critically depends on a precisely regulated spatiotemporal sequence of highly dynamic and plastic chromatin rearrangements, which influence the access of repair proteins to damage sites, interaction between repeats, and other phenomena closely related to HR efficiency and fidelity.

On the other hand, NHEJ may be relatively precise even in repeat-rich chromatin terrain, unless the chromatin is extensively fragmented. Decondensation of heterochromatin, which is necessary for the formation of repair complexes within both the HR and NHEJ repair pathways, also protects against illegitimate recombination, while in NHEJ, it exacerbates the risk of interactions between DSBs.

Mutual interactions between the incident radiation and local chromatin architecture further modify the micro- and nanoarchitecture of the damage site and, in turn, the potential risk of damage misrepair (summarized in Table 1). Correct selection of the best repair pathway under given pancellular and site-specific conditions is therefore nontrivial and directly influences the fate of damaged cells [9,17]. Hence, we conclude this review with a brief discussion on the relationship between the physical parameters of the ionizing radiation, the chromatin architecture, and the formation mechanism of chromosomal aberrations, representing common precursors of cancer development. As comprehensive coverage of all mutation and aberration types potentially resulting from DSB misrepair goes far beyond the scope of this review, only chromosomal deletions and structural aberrations are considered as illustrative examples.

Highly localized energy deposition by high-LET radiation causes extensive local chromatin fragmentation. As Ku proteins can bind to these fragments only with difficulty, HR appears to be the dominant repair pathway for high-LET-induced damage in G2 cells. In G1 cells, or in cells where the HR machinery (e.g., the BRCA2 or RAD51 protein) is exhausted upon, for instance, high radiation doses, alternative repair pathways (i.e., A-Ej) may be activated (reviewed in [13,174]). Upon high-LET irradiation, DSB clusters (“primary clusters” [50]) are formed directly as a consequence of dense ionization events. It is not difficult to imagine that these clusters, with their many adjacent free DNA ends, constitute an ideal substrate for the easy formation of complex chromosomal translocations and other aberrations that cause the large diversity of individual cancer genomes, similar to chromothripsis [175,176]. As high-LET radiation may greatly disrupt the integrity of heterochromatin domains and unmask DNA repeats, HR may become deregulated or replaced by SSA, ending with the generation of large deletions. Indeed, when repetitive sequences were inserted in plasmids, SSA—rather than classical HR—was observed [177]. In addition, the greater the LET of the radiation and complexity of the DSB lesions, the higher is the proportion of intrachromosomal aberrations compared to interchromosomal aberrations. [178,179].

In contrast, sparsely ionizing radiation distributes its energy in the nucleus much more homogeneously; thus, very large focus clusters (or even focus cluster traces) with fragmented chromatin do not form. Therefore, the individual foci are, in most cases, relatively distant from each other. This situation reduces the risk of DNA end misrejoining, and the NHEJ pathway thus seems to be generally preferred and an acceptably precise repair mechanism in euchromatin. On the other hand, the dense and complex architecture of heterochromatin requires HR (or NHEJ, if HR is not available) coupled with the ATM signaling pathway and chromatin decondensation [36]. In contrast to the severe disruption of heterochromatin domains seen in cells exposed to high-LET radiation, heterochromatin domain architecture remains preserved in cells exposed to low-LET photonic rays. Hence, except for the promotion of short deletions/insertions by NHEJ, both the NHEJ and HR pathways seem to operate with relatively high precision.

However, due to its extensive architectural rearrangement during repair, irradiated heterochromatin may be especially prone to not only mutations but also epimutations, i.e., permanent epigenetic/structural alterations at damage sites with functional consequences [57,180,181,182,183]. The risk of epimutations can be expected to correlate with the architecture of damaged chromatin domains rather than the physical parameters of the incident radiation; however, this phenomenon remains to be studied.

Complex chromosomal translocations may occur even in cells irradiated with photon radiation, indicating that local chromatin architecture rearrangements might also induce global chromatin reorganization, as can be observed in carcinogenesis [75,130,184]. Therefore, how do all these translocations arise? Previously, two seemingly conflicting hypotheses were formulated to explain this phenomenon (reviewed in [48]). The first model (the “position-first hypothesis”) [48,185,186] considers DSBs to be generally immobile and assumes that chromatin interchanges can occur only between genomic loci already located near each other before irradiation. However, this hypothesis cannot explain how translocations between relatively distant loci in the nucleus or even complex translocations involving several chromosomes occur under the given conditions of immobile DSBs. 

The second model (the “breakage-first hypothesis”) [48,186] basically contradicts the position-first hypothesis, since it envisages dynamic migration of DSBs to several centers, so-called repair factories, where several DSBs are repaired together. This, in principle, resembles the situation in which primary DSB clusters form after exposure to densely ionizing radiation. However, although the breakage-first hypothesis elegantly explains the origin of translocations between mutually distant and possibly multiple loci, complex translocations probably occur much more often than actually observed.

It is said that the truth is always somewhere in the middle, and this also seems to be true regarding the mechanism of chromosomal translocation formation [75] (reviewed in [48]). Our measurements of total IRIF mobility in **γ**-irradiated cells did not reveal any significant differences from intact chromatin; however, an IRIF fraction was discovered that exhibited significantly higher mobility [75]. Movements of these IRIFs, though limited in space, often resulted in the formation of clusters of two, three or occasionally more IRIF foci. The reason for this surprising behavior was subsequently identified to be decondensation of damaged heterochromatin domains [75]. Indeed, many later studies (e.g., [77,93,187,188,189]) confirmed that chromatin decondensation must precede DSB repair in heterochromatin, probably to allow the assembly of repair complexes at heterochromatic DSB sites [77]. Decondensation of heterochromatin often leads to the protrusion of IRIFs into cell nucleus regions with low-density chromatin. However, due to the limited volume of these spaces (“chromatin holes”), collisions between IRIFs occasionally occur, especially after exposure to high radiation doses. Emerging aggregates may be temporary in nature, but they sometimes become stable clusters, which we call secondary clusters [50] to emphasize that they appear as a byproduct of DNA repair and not directly as a result of radiation energy deposition. Clustering during repair as described above and heterochromatin rearrangements can thus be interpreted as a result of DSB reorganization/trafficking, which is an integral part of the repair mechanism.

In summary, our hybrid model of translocation formation combines certain aspects of the position-first and breakage-first hypotheses and emphasizes the role of chromatin micro- [75] and nanoarchitecture [120] in this process and in cancer induction and development. Most DSBs are generally stable in space and are repaired separately at the sites of their origin. However, DSBs localized in heterochromatin become more motile due to decondensation of the damaged domain driven by repair processes [75] and eventually form secondary IRIF clusters, generating substrates for illegitimate chromatin exchanges associated with a risk of gene malfunction and further global chromatin rearrangements during cell division. The formation of secondary DSB clusters thus potentially explains how complex translocations and translocations between mutually distant loci can occur occasionally even in cells damaged by sparsely ionizing radiation. Our newly proposed hybrid model also clarifies why complex translocations generated by densely and sparsely ionizing radiation differ so greatly in the number of participating DSBs and why exposure to densely ionizing radiation efficiently elicits intrachromosomal aberrations. Hence, the mechanism of chromosomal translocation formation seems to be quite different for photon and high-LET particle radiation, but both may induce global changes in chromatin architecture and function.

A widely accepted paradigm in radiobiology is that translocations occur preferentially between spatially adjacent genetic loci, as supported, for instance, by the high prevalence of spontaneously formed leukemogenic translocations in accordance with this rule [185,190,191,192,193] and by experiments with high-LET radiation (e.g., [193]). However, our hybrid model provides strong evidence that the chromatin architecture around a DSB may affect the probability of translocation between particular loci more strongly than the mutual distance between the loci in the nucleus if this distance is not large enough to be dominant [75]. As the functional architecture of chromatin determines both the nuclear gene topology [44,45,46,130,194,195,196,197] and the vectors (extent and directions) of individual DSB movements relative to each other, it also defines the probability of mutual DSB interaction [48,75].

In addition, by comparing IRIF formation in structurally and functionally distinct chromatin domains labeled with domain-specific DNA probes, we showed that photon radiation preferentially damages active genes, probably due to the open architecture and decreased abundance of proteins in euchromatin [76]. Similarly, fivefold to 50-fold fewer DSBs than in physiological chromatin domains were observed in irradiated cells with various degrees of chromatin condensation adjusted by different concentrations of Mg^2+^ ions [198]. Taken together, these observations indicate that chromatin architecture and its interaction with ionizing radiation with given physical characteristics critically influence the mechanism and accuracy of DSB repair, thereby influencing the risk and type of genetic defects (mutations and chromosomal aberrations) and the processes of cancer induction and development in multiple ways.

## 6. Conclusions and Future Perspectives

The importance of architectural features in DNA damage and repair has long been overlooked due to the lack of superresolution light microscopy technologies. During the last decade, numerous microscopy studies indisputably demonstrated that like other processes in the cell nucleus—transcription and replication (see, for instance, [199] and citations therein)—DSB repair is strongly influenced by chromatin architecture and *vice versa*. This relationship probably also holds true for the decision-making step for a particular repair pathway at each individual DSB site [127,187]. In addition, chromatin architecture plays multiple roles in determining both chromatin sensitivity to radiation damage [76,200] and DSB repair mechanisms, including the risk of chromosomal aberrations [75]. These conclusions built on standard optical confocal microscopy studies have recently been supported by several nanoscale studies, which have become possible thanks to the remarkable development of superresolution microscopy techniques.

Obviously, the results outlined in the present review and the enormous biochemical complexity of DNA repair (only ATM phosphorylates more than 700 targets [201]) imply the existence of a certain overarching mechanism that helps to spatiotemporally organize all (biochemical) repair processes. Moreover, the human genome is organized into structurally and functionally distinct chromatin domains, each type of which may have specific requirements for repair. Based on these considerations and the current literature, it can be assumed that the architecture of the affected chromatin domain, the character of the DSB, and the initiated repair mechanism at the site are integrally imprinted in the IRIF architecture. The IRIF architecture, in turn, participates in multiple steps of DSB repair control [128]. However, drawing conclusions about a causal relationship between the architecture of chromatin and IRIFs and the mechanism by which cells activate a particular repair pathway at a particular DSB site remains difficult. Entirely new insights into this scientific content are, in the foreseeable future, expected from microscopy and nanoscopy studies correlating the micro-, meso-, and nanoscale aspects of chromatin and IRIF architecture. SMLM is a very promising technique in this regard since it bridges the resolution gap between confocal fluorescence microscopy and electron microscopy. Moreover, the novel specimen preparation technique for SMLM allows the same cell specimen to be used in-line for all three microscopic systems (confocal light microscopy - SMLM -> electron microscopy) [202].

In conclusion, the architectural features of chromatin and IRIFs play important roles in the regulation of DSB repair, as they help the repair machinery to escape from the repair-restrictive environment of chromatin and create an optimal environment for a particular repair mechanism [81]. Newly emerging correlated multiscale structuromics may soon revolutionize our understanding of DSB repair pathways. However, currently, the DSB repair system is increasingly being recognized to work as a dynamic network rather than as isolated pathways. As demonstrated by recent studies, NHEJ operating in heterochromatin or on DSBs that are difficult to repair exhibits aspects of both cNHEJ and HR [160]. Additionally, alternative repair pathways may be active even when cNHEJ and HR are available [11]. The interconnection of DSB repair processes into a network thus allows fine-tuning of repair at individual damage sites, the mechanisms of which oscillate between the extremes of NHEJ and HR. This scenario emphasizes the relevance of factors regulating local DSB repair but complicates our research on DSB repair control.

## Figures and Tables

**Table 1:** Relationship between the physical characteristics of the incident radiation, the chromatin architecture at the DSB damage site, the utilized repair mechanism (pathway) and the types of potentially generated chromosomal aberrations or epimutations.

Repair Pathway	Chromatin Domain Type (Architecture and Function)			
	Euchromatin		Heterochromatin	
**cNHEJ**	short deletions (due to DNA-end "cleaning") interchromosomal translocations and other aberration types	x	x	x
**ATM-NHEJ**	x	x	short deletions (due to DNA-end "cleaning") interchromosomal translocations and other aberration types (compared to NHEJ, chromatin decondensation increases the risk of aberrations but, at the same time, Hc is more resistant to DSB induction than Ec) epimutations	x
**HR/RNA-HR**	highly precise	highly precise	x	x
**ATM-HR**	x	x	epimutations	large deletions complex translocations (due to "premature" aberrant recombination between unmasked repeats in disintegrated domains), interchromosomal aberration types epimutations
**Alternative pathways** **(A-Ej)**	large deletions translocations (both due to principal interaction bentween repeats)	large deletions complex translocations (both due to principal interraction bentween repeats and chromatin fragmentation) interchromosomal aberration types	large deletions translocations (both due to principal interraction bentween repeats) epimutations	large deletions complex translocations (both due to principal recombination between repeats and chromatin fragmentation; for a higher repeat content in Hc, more serious damage than in Ec) interchromosomal aberration types epimutations
	**Low-LET**	**High-LET**	**Low-LET**	**High-LET**
	**Radiation quality**			

Ec: euchromatin, Hc: heterochromatin, Low-LET: ionizing radiation with low linear energy transfer, High-LET: ionizing radiation with high linear energy transfer, X: repair pathway less relevant or irrelevant under the given condition (factor combination). Processes responsible for the generation of particular aberration types are indicated in brackets.

## Abbreviations

A-Ej	alternative end joining mechanisms (alternative repair pathways)
cNHEJ	classical nonhomologous end joining (dependent on Ku70/80)
DSB	double-strand break
High-LET	high linear energy transfer
HR	homologous recombination
H3K9me3	histone H3 trimethylated at lysine 9
H3K36me3	histone H3 trimethylated at lysine 39
H4K20me3	histone H4 trimethylated at lysine 20
IRIF	ionizing radiation-induced focus/DSB repair complexes
IR	ionizing radiation
Low-LET	low linear energy transfer
MMEJ	microhomology-mediated end joining
NHEJ	nonhomologous end joining
SMLM	single-molecule localization microscopy
γH2AX	histone H2AX phosphorylated on serine 139

## References

[1] Alhmoud J.F., Woolley J.F., Al Moustafa A.-E., Malki M.I. (2020). DNA Damage/Repair Management in Cancers. Cancers.

[2] Chatterjee N., Walker G.C. (2017). Mechanisms of DNA damage, repair, and mutagenesis: DNA Damage and Repair. Environ. Mol. Mutagen..

[3] Rittich B., Spanová A., Falk M., Benes M.J., Hrubý M. (2004). Cleavage of double stranded plasmid DNA by lanthanide complexes. J. Chromatogr. B Anal. Technol. Biomed. Life Sci..

[4] Bennett C.B., Lewis A.L., Baldwin K.K., Resnick M.A. (1993). Lethality induced by a single site-specific double-strand break in a dispensable yeast plasmid. Proc. Natl. Acad. Sci. USA.

[5] Sharda A., Rashid M., Shah S.G., Sharma A.K., Singh S.R., Gera P., Chilkapati M.K., Gupta S. (2020). Elevated HDAC activity and altered histone phospho-acetylation confer acquired radio-resistant phenotype to breast cancer cells. Clin. Epigenetics.

[6] Ensminger M., Löbrich M. (2020). One end to rule them all: Non-homologous end-joining and homologous recombination at DNA double-strand breaks. Br. J. Radiol..

[7] Scully R., Panday A., Elango R., Willis N.A. (2019). DNA double-strand break repair-pathway choice in somatic mammalian cells. Nat. Rev. Mol. Cell Biol..

[8] Sallmyr A., Tomkinson A.E. (2018). Repair of DNA double-strand breaks by mammalian alternative end-joining pathways. J. Biol. Chem..

[9] Chang H.H.Y., Pannunzio N.R., Adachi N., Lieber M.R. (2017). Non-homologous DNA end joining and alternative pathways to double-strand break repair. Nat. Rev. Mol. Cell Biol..

[10] Iliakis G., Murmann T., Soni A. (2015). Alternative end-joining repair pathways are the ultimate backup for abrogated classical non-homologous end-joining and homologous recombination repair: Implications for the formation of chromosome translocations. Mutat. Res. Genet. Toxicol. Environ. Mutagen..

[11] Xiong X., Du Z., Wang Y., Feng Z., Fan P., Yan C., Willers H., Zhang J. (2015). 53BP1 promotes microhomology-mediated end-joining in G1-phase cells. Nucleic Acids Res..

[12] Iliakis G. (2009). Backup pathways of NHEJ in cells of higher eukaryotes: Cell cycle dependence. Radiother. Oncol..

[13] Iliakis G., Mladenov E., Mladenova V. (2019). Necessities in the Processing of DNA Double Strand Breaks and Their Effects on Genomic Instability and Cancer. Cancers.

[14] Verma P., Greenberg R.A. (2016). Noncanonical views of homology-directed DNA repair. Genes Dev..

[15] Vanoli F., Prakash R., White T., Jasin M., Aguilera A., Carreira A. (2021). Interhomolog Homologous Recombination in Mouse Embryonic Stem Cells. Homologous Recombination.

[16] Fernandez J., Bloomer H., Kellam N., LaRocque J.R. (2019). Chromosome Preference During Homologous Recombination Repair of DNA Double-Strand Breaks in *Drosophila melanogaster*. Genes Genomes Genet..

[17] Campos A., Clemente-Blanco A. (2020). Cell Cycle and DNA Repair Regulation in the Damage Response: Protein Phosphatases Take Over the Reins. IJMS.

[18] Hustedt N., Durocher D. (2017). The control of DNA repair by the cell cycle. Nat. Cell Biol..

[19] You Z., Bailis J.M. (2010). DNA damage and decisions: CtIP coordinates DNA repair and cell cycle checkpoints. Trends Cell Biol..

[20] Bordelet H., Dubrana K. (2019). Keep moving and stay in a good shape to find your homologous recombination partner. Curr. Genet..

[21] Katsuki Y., Jeggo P.A., Uchihara Y., Takata M., Shibata A. (2020). DNA double-strand break end resection: A critical relay point for determining the pathway of repair and signaling. Genome Instab. Dis..

[22] Choi E.-H., Yoon S., Koh Y.E., Seo Y.-J., Kim K.P. (2020). Maintenance of genome integrity and active homologous recombination in embryonic stem cells. Exp. Mol. Med..

[23] Kohutova A., Raška J., Kruta M., Seneklova M., Barta T., Fojtik P., Jurakova T., Walter C.A., Hampl A., Dvorak P. (2019). Ligase 3–mediated end-joining maintains genome stability of human embryonic stem cells. FASEB J..

[24] Shrivastav M., De Haro L.P., Nickoloff J.A. (2008). Regulation of DNA double-strand break repair pathway choice. Cell Res..

[25] Serrano L., Liang L., Chang Y., Deng L., Maulion C., Nguyen S., Tischfield J.A. (2011). Homologous Recombination Conserves DNA Sequence Integrity Throughout the Cell Cycle in Embryonic Stem Cells. Stem Cells Dev..

[26] Mujoo K., Pandita R.K., Tiwari A., Charaka V., Chakraborty S., Singh D.K., Hambarde S., Hittelman W.N., Horikoshi N., Hunt C.R. (2017). Differentiation of Human Induced Pluripotent or Embryonic Stem Cells Decreases the DNA Damage Repair by Homologous Recombination. Stem Cell Rep..

[27] Lukášová E., Kořistek Z., Klabusay M., Ondřej V., Grigoryev S., Bačíková A., Řezáčová M., Falk M., Vávrová J., Kohútová V. (2013). Granulocyte maturation determines ability to release chromatin NETs and loss of DNA damage response; these properties are absent in immature AML granulocytes. Biochim. Biophys. Acta.

[28] Jeggo P.A., Pearl L.H., Carr A.M. (2016). DNA repair, genome stability and cancer: A historical perspective. Nat. Rev. Cancer.

[29] Dietlein F., Thelen L., Reinhardt H.C. (2014). Cancer-specific defects in DNA repair pathways as targets for personalized therapeutic approaches. Trends Genet..

[30] Ma J., Setton J., Lee N.Y., Riaz N., Powell S.N. (2018). The therapeutic significance of mutational signatures from DNA repair deficiency in cancer. Nat. Commun..

[31] Delabaere L., Ertl H.A., Massey D.J., Hofley C.M., Sohail F., Bienenstock E.J., Sebastian H., Chiolo I., LaRocque J.R. (2017). Aging impairs double-strand break repair by homologous recombination in *Drosophila* germ cells. Aging Cell.

[32] Bristow R.G., Hill R.P. (2008). Hypoxia, DNA repair and genetic instability. Nat. Rev. Cancer.

[33] Carreau A., Hafny-Rahbi B.E., Matejuk A., Grillon C., Kieda C. (2011). Why is the partial oxygen pressure of human tissues a crucial parameter? Small molecules and hypoxia. J. Cell. Mol. Med..

[34] Hagiwara Y., Oike T., Niimi A., Yamauchi M., Sato H., Limsirichaikul S., Held K.D., Nakano T., Shibata A. (2019). Clustered DNA double-strand break formation and the repair pathway following heavy-ion irradiation. J. Radiat. Res..

[35] Nogueira A., Fernandes M., Catarino R., Medeiros R. (2019). RAD52 Functions in Homologous Recombination and Its Importance on Genomic Integrity Maintenance and Cancer Therapy. Cancers.

[36] Löbrich M., Jeggo P. (2017). A Process of Resection-Dependent Nonhomologous End Joining Involving the Goddess Artemis. Trends Biochem. Sci..

[37] Kakarougkas A., Jeggo P.A. (2014). DNA DSB repair pathway choice: An orchestrated handover mechanism. Br. J. Radiol..

[38] Bader A.S., Hawley B.R., Wilczynska A., Bushell M. (2020). The roles of RNA in DNA double-strand break repair. Br. J. Cancer.

[39] Ingram S.P., Warmenhoven J.W., Henthorn N.T., Smith E.A.K., Chadwick A.L., Burnet N.G., Mackay R.I., Kirkby N.F., Kirkby K.J., Merchant M.J. (2019). Mechanistic modelling supports entwined rather than exclusively competitive DNA double-strand break repair pathway. Sci. Rep..

[40] Jezkova L., Zadneprianetc M., Kulikova E., Smirnova E., Bulanova T., Depes D., Falkova I., Boreyko A., Krasavin E., Davidkova M. (2018). Particles with similar LET values generate DNA breaks of different complexity and reparability: A high-resolution microscopy analysis of γH2AX/53BP1 foci. Nanoscale.

[41] Schipler A., Iliakis G. (2013). DNA double-strand-break complexity levels and their possible contributions to the probability for error-prone processing and repair pathway choice. Nucleic Acids Res..

[42] Cruz G.A.S. (2016). Microdosimetry: Principles and applications. Rep. Pract. Oncol. Radiother..

[43] Hofmann W., Li W.B., Friedland W., Miller B.W., Madas B., Bardiès M., Balásházy I. (2020). Internal microdosimetry of alpha-emitting radionuclides. Radiat. Environ. Biophys..

[44] Kozubek S., Lukásová E., Jirsová P., Koutná I., Kozubek M., Ganová A., Bártová E., Falk M., Paseková R. (2002). 3D Structure of the human genome: Order in randomness. Chromosoma.

[45] Cremer T., Cremer M., Hübner B., Strickfaden H., Smeets D., Popken J., Sterr M., Markaki Y., Rippe K., Cremer C. (2015). The 4D nucleome: Evidence for a dynamic nuclear landscape based on co-aligned active and inactive nuclear compartments. FEBS Lett..

[46] Lukásová E., Kozubek S., Kozubek M., Falk M., Amrichová J. (2002). The 3D structure of human chromosomes in cell nuclei. Chromosome Res..

[47] Crosetto N., Bienko M. (2020). Radial Organization in the Mammalian Nucleus. Front. Genet..

[48] Falk M., Lukasova E., Kozubek S. (2010). Higher-order chromatin structure in DSB induction, repair and misrepair. Mutat. Res..

[49] Falk M., Hausmann M., Lukášová E., Biswas A., Hildenbrand G., Davídková M., Krasavin E., Kleibl Z., Falková I., Ježková L. (2014). Determining Omics spatiotemporal dimensions using exciting new nanoscopy techniques to assess complex cell responses to DNA damage: Part A—Radiomics. Crit. Rev. Eukaryot. Gene Expr..

[50] Falk M., Hausmann M., Lukášová E., Biswas A., Hildenbrand G., Davídková M., Krasavin E., Kleibl Z., Falková I., Ježková L. (2014). Determining Omics spatiotemporal dimensions using exciting new nanoscopy techniques to assess complex cell responses to DNA damage: Part B—Structuromics. Crit. Rev. Eukaryot. Gene Expr..

[51] Zhao L., Bao C., Shang Y., He X., Ma C., Lei X., Mi D., Sun Y. (2020). The Determinant of DNA Repair Pathway Choices in Ionising Radiation-Induced DNA Double-Strand Breaks. Biomed Res. Int..

[52] Ruebe C.E., Lorat Y., Schanz S., Schuler N., Ruebe C. (2011). DNA Double-strand Break Repair in the Context of Chromatin. Int. J. Radiat. Oncol. Biol. Phys..

[53] Shibata A. (2017). Regulation of repair pathway choice at two-ended DNA double-strand breaks. Mutat. Res. Fundam. Mol. Mech. Mutagenesis.

[54] Clouaire T., Legube G. (2015). DNA double strand break repair pathway choice: A chromatin based decision?. Nucleus.

[55] Goodarzi A.A., Jeggo P.A. (2012). The Heterochromatic Barrier to DNA Double Strand Break Repair: How to Get the Entry Visa. IJMS.

[56] Noon A.T., Shibata A., Rief N., Löbrich M., Stewart G.S., Jeggo P.A., Goodarzi A.A. (2010). 53BP1-dependent robust localized KAP-1 phosphorylation is essential for heterochromatic DNA double-strand break repair. Nat. Cell Biol..

[57] Ježková L., Falk M., Falková I., Davídková M., Bačíková A., Štefančíková L., Vachelová J., Michaelidesová A., Lukášová E., Boreyko A. (2014). Function of chromatin structure and dynamics in DNA damage, repair and misrepair: γ-rays and protons in action. Appl. Radiat. Isot..

[58] Lemaître C., Grabarz A., Tsouroula K., Andronov L., Furst A., Pankotai T., Heyer V., Rogier M., Attwood K.M., Kessler P. (2014). Nuclear position dictates DNA repair pathway choice. Genes Dev..

[59] Her J., Bunting S.F. (2018). How cells ensure correct repair of DNA double-strand breaks. J. Biol. Chem..

[60] Brinkman E.K., Chen T., de Haas M., Holland H.A., Akhtar W., van Steensel B. (2018). Kinetics and Fidelity of the Repair of Cas9-Induced Double-Strand DNA Breaks. Mol. Cell.

[61] Cannan W.J., Pederson D.S. (2016). Mechanisms and Consequences of Double-Strand DNA Break Formation in Chromatin: Double-Strand Dna Break Formation in Chromatin. J. Cell. Physiol..

[62] Nickoloff J.A., Sharma N., Taylor L. (2020). Clustered DNA Double-Strand Breaks: Biological Effects and Relevance to Cancer Radiotherapy. Genes.

[63] Mavragani I.V., Nikitaki Z., Kalospyros S.A., Georgakilas A.G. (2019). Ionizing Radiation and Complex DNA Damage: From Prediction to Detection Challenges and Biological Significance. Cancers.

[64] Osipov A.N., Grekhova A., Pustovalova M., Ozerov I.V., Eremin P., Vorobyeva N., Lazareva N., Pulin A., Zhavoronkov A., Roumiantsev S. (2015). Activation of homologous recombination DNA repair in human skin fibroblasts continuously exposed to X-ray radiation. Oncotarget.

[65] Somaiah N., Yarnold J., Lagerqvist A., Rothkamm K., Helleday T. (2013). Homologous recombination mediates cellular resistance and fraction size sensitivity to radiation therapy. Radiother. Oncol..

[66] Salles B. (2013). Inhibition of the non homologous end joining process in the context of hypoxic tumor cells. Transl. Cancer Res..

[67] Cowman S., Pizer B., Sée V. (2020). Downregulation of both mismatch repair and non-homologous end-joining pathways in hypoxic brain tumour cell lines. bioRxiv.

[68] Redon C.E., Dickey J.S., Bonner W.M., Sedelnikova O.A. (2009). γ-H2AX as a biomarker of DNA damage induced by ionizing radiation in human peripheral blood lymphocytes and artificial skin. Adv. Space Res..

[69] Rogakou E.P., Boon C., Redon C., Bonner W.M. (1999). Megabase chromatin domains involved in DNA double-strand breaks in vivo. J. Cell Biol..

[70] Paull T.T. (2015). Mechanisms of ATM Activation. Annu. Rev. Biochem..

[71] Podhorecka M., Skladanowski A., Bozko P. (2010). H2AX Phosphorylation: Its Role in DNA Damage Response and Cancer Therapy. J. Nucleic Acids.

[72] Bekker-Jensen S., Mailand N. (2010). Assembly and function of DNA double-strand break repair foci in mammalian cells. DNA Repair.

[73] Vítor A.C., Huertas P., Legube G., de Almeida S.F. (2020). Studying DNA Double-Strand Break Repair: An Ever-Growing Toolbox. Front. Mol. Biosci..

[74] Hofer M., Falk M., Komůrková D., Falková I., Bačíková A., Klejdus B., Pagáčová E., Štefančíková L., Weiterová L., Angelis K.J. (2016). Two New Faces of Amifostine: Protector from DNA Damage in Normal Cells and Inhibitor of DNA Repair in Cancer Cells. J. Med. Chem..

[75] Falk M., Lukasova E., Gabrielova B., Ondrej V., Kozubek S. (2007). Chromatin dynamics during DSB repair. Biochim. Biophys. Acta.

[76] Falk M., Lukásová E., Kozubek S. (2008). Chromatin structure influences the sensitivity of DNA to gamma-radiation. Biochim. Biophys. Acta.

[77] Falk M., Lukášová E., Štefančíková L., Baranová E., Falková I., Ježková L., Davídková M., Bačíková A., Vachelová J., Michaelidesová A. (2014). Heterochromatinization associated with cell differentiation as a model to study DNA double strand break induction and repair in the context of higher-order chromatin structure. Appl. Radiat. Isot..

[78] Belyaev I.Y. (2010). Radiation-induced DNA repair foci: Spatio-temporal aspects of formation, application for assessment of radiosensitivity and biological dosimetry. Mutat. Res. Rev. Mutat. Res..

[79] Goodarzi A.A., Jeggo P.A. (2012). Irradiation induced foci (IRIF) as a biomarker for radiosensitivity. Mutat. Res. Fundam. Mol. Mech. Mutagenesis.

[80] Rothkamm K., Barnard S., Moquet J., Ellender M., Rana Z., Burdak-Rothkamm S. (2015). DNA damage foci: Meaning and significance. Environ. Mol. Mutagen..

[81] Tonnemacher S., Eltsov M., Jakob B. (2020). Correlative Light and Electron Microscopy (CLEM) Analysis of Nuclear Reorganization Induced by Clustered DNA Damage Upon Charged Particle Irradiation. IJMS.

[82] Entrz Pubmed Database. https://pubmed.ncbi.nlm.nih.gov.

[83] Falk M., Hausmann M. (2020). Advances in research of DNA damage and repair in cells exposed to various types of ionizing radiation in the era of super-resolution optical microscopy. Cas Lek Cesk.

[84] Fabre E., Zimmer C. (2018). From dynamic chromatin architecture to DNA damage repair and back. Nucleus.

[85] Arnould C., Legube G. (2020). The Secret Life of Chromosome Loops upon DNA Double-Strand Break. J. Mol. Biol..

[86] Jeggo P.A., Downs J.A. (2014). Roles of chromatin remodellers in DNA double strand break repair. Exp. Cell Res..

[87] Watts F. (2016). Repair of DNA Double-Strand Breaks in Heterochromatin. Biomolecules.

[88] Caron H., van Schaik B., van der Mee M., Baas F., Riggins G., van Sluis P., Hermus M.C., van Asperen R., Boon K., Voûte P.A. (2001). The human transcriptome map: Clustering of highly expressed genes in chromosomal domains. Science.

[89] Versteeg R. (2003). The Human Transcriptome Map Reveals Extremes in Gene Density, Intron Length, GC Content, and Repeat Pattern for Domains of Highly and Weakly Expressed Genes. Genome Res..

[90] Goodarzi A.A., Noon A.T., Jeggo P.A. (2009). The impact of heterochromatin on DSB repair. Biochem. Soc. Trans..

[91] Goodarzi A.A., Jeggo P., Lobrich M. (2010). The influence of heterochromatin on DNA double strand break repair: Getting the strong, silent type to relax. DNA Repair.

[92] Baldock R.A., Day M., Wilkinson O.J., Cloney R., Jeggo P.A., Oliver A.W., Watts F.Z., Pearl L.H. (2015). ATM Localization and Heterochromatin Repair Depend on Direct Interaction of the 53BP1-BRCT2 Domain with γH2AX. Cell Rep..

[93] Goodarzi A.A., Noon A.T., Deckbar D., Ziv Y., Shiloh Y., Löbrich M., Jeggo P.A. (2008). ATM signaling facilitates repair of DNA double-strand breaks associated with heterochromatin. Mol. Cell.

[94] Fortuny A., Polo S.E. (2018). The response to DNA damage in heterochromatin domains. Chromosoma.

[95] Walter A., Chapuis C., Huet S., Ellenberg J. (2013). Crowded chromatin is not sufficient for heterochromatin formation and not required for its maintenance. J. Struct. Biol..

[96] Jakob B., Splinter J., Conrad S., Voss K.-O., Zink D., Durante M., Löbrich M., Taucher-Scholz G. (2011). DNA double-strand breaks in heterochromatin elicit fast repair protein recruitment, histone H2AX phosphorylation and relocation to euchromatin. Nucleic Acids Res..

[97] Svetličič M., Bomhard A., Sterr C., Brückner F., Płódowska M., Lisowska H., Lundholm L. (2020). Alpha Radiation as a Way to Target Heterochromatic and Gamma Radiation-Exposed Breast Cancer Cells. Cells.

[98] Yu S., Yang F., Shen W.H. (2016). Genome maintenance in the context of 4D chromatin condensation. Cell. Mol. Life Sci..

[99] Chiolo I., Minoda A., Colmenares S.U., Polyzos A., Costes S.V., Karpen G.H. (2011). Double-Strand Breaks in Heterochromatin Move Outside of a Dynamic HP1a Domain to Complete Recombinational Repair. Cell.

[100] Luijsterburg M.S., van Attikum H. (2012). Close encounters of the RNF8th kind: When chromatin meets DNA repair. Curr. Opin. Cell Biol..

[101] Amaral N., Ryu T., Li X., Chiolo I. (2017). Nuclear Dynamics of Heterochromatin Repair. Trends Genet..

[102] Foltánková V., Legartová S., Kozubek S., Hofer M., Bártová E. (2013). DNA-damage response in chromatin of ribosomal genes and the surrounding genome. Gene.

[103] Lorat Y., Timm S., Jakob B., Taucher-Scholz G., Rübe C.E. (2016). Clustered double-strand breaks in heterochromatin perturb DNA repair after high linear energy transfer irradiation. Radiother. Oncol..

[104] Timm S., Lorat Y., Jakob B., Taucher-Scholz G., Rübe C.E. (2018). Clustered DNA damage concentrated in particle trajectories causes persistent large-scale rearrangements in chromatin architecture. Radiother. Oncol..

[105] Zhang Y., Heermann D.W. (2014). DNA double-strand breaks: Linking gene expression to chromosome morphology and mobility. Chromosoma.

[106] Hausmann M., Wagner E., Lee J.-H., Schrock G., Schaufler W., Krufczik M., Papenfuß F., Port M., Bestvater F., Scherthan H. (2018). Super-resolution localization microscopy of radiation-induced histone H2AX-phosphorylation in relation to H3K9-trimethylation in HeLa cells. Nanoscale.

[107] Zhang Y., Máté G., Müller P., Hillebrandt S., Krufczik M., Bach M., Kaufmann R., Hausmann M., Heermann D.W. (2015). Radiation induced chromatin conformation changes analysed by fluorescent localization microscopy, statistical physics, and graph theory. PLoS ONE.

[108] Aymard F., Bugler B., Schmidt C.K., Guillou E., Caron P., Briois S., Iacovoni J.S., Daburon V., Miller K.M., Jackson S.P. (2014). Transcriptionally active chromatin recruits homologous recombination at DNA double-strand breaks. Nat. Struct. Mol. Biol..

[109] Aymard F., Aguirrebengoa M., Guillou E., Javierre B.M., Bugler B., Arnould C., Rocher V., Iacovoni J.S., Biernacka A., Skrzypczak M. (2017). Genome-wide mapping of long-range contacts unveils clustering of DNA double-strand breaks at damaged active genes. Nat. Struct. Mol. Biol..

[110] Deckbar D., Jeggo P.A., Löbrich M. (2011). Understanding the limitations of radiation-induced cell cycle checkpoints. Crit. Rev. Biochem. Mol. Biol..

[111] Chao H.X., Poovey C.E., Privette A.A., Grant G.D., Chao H.Y., Cook J.G., Purvis J.E. (2017). Orchestration of DNA Damage Checkpoint Dynamics across the Human Cell Cycle. Cell Syst..

[112] Chakraborty A., Tapryal N., Venkova T., Horikoshi N., Pandita R.K., Sarker A.H., Sarkar P.S., Pandita T.K., Hazra T.K. (2016). Classical non-homologous end-joining pathway utilizes nascent RNA for error-free double-strand break repair of transcribed genes. Nat. Commun..

[113] Domingo-Prim J., Bonath F., Visa N. (2020). RNA at DNA Double-Strand Breaks: The Challenge of Dealing with DNA: RNA Hybrids. BioEssays.

[114] Monajembashi S., Rapp A., Schmitt E., Dittmar H., Greulich K.-O., Hausmann M. (2005). Spatial Association of Homologous Pericentric Regions in Human Lymphocyte Nuclei during Repair. Biophys. J..

[115] Janssen A., Colmenares S.U., Lee T., Karpen G.H. (2019). Timely double-strand break repair and pathway choice in pericentromeric heterochromatin depend on the histone demethylase dKDM4A. Genes Dev..

[116] Fyodorov D.V., Zhou B.-R., Skoultchi A.I., Bai Y. (2018). Emerging roles of linker histones in regulating chromatin structure and function. Nat. Rev. Mol. Cell Biol..

[117] Falk M., Falková I., Kopečná O., Bačíková A., Pagáčová E., Šimek D., Golan M., Kozubek S., Pekarová M., Follett S.E. (2018). Chromatin architecture changes and DNA replication fork collapse are critical features in cryopreserved cells that are differentially controlled by cryoprotectants. Sci. Rep..

[118] Caron P., van der Linden J., van Attikum H. (2019). Bon voyage: A transcriptional journey around DNA breaks. DNA Repair.

[119] Kalousi A., Soutoglou E. (2016). Nuclear compartmentalization of DNA repair. Curr. Opin. Genet. Dev..

[120] Bobkova E., Depes D., Lee J.-H., Jezkova L., Falkova I., Pagacova E., Kopecna O., Zadneprianetc M., Bacikova A., Kulikova E. (2018). Recruitment of 53BP1 Proteins for DNA Repair and Persistence of Repair Clusters Differ for Cell Types as Detected by Single Molecule Localization Microscopy. Int. J. Mol. Sci..

[121] Schwarz B., Friedl A.A., Girst S., Dollinger G., Reindl J. (2019). Nanoscopic analysis of 53BP1, BRCA1 and Rad51 reveals new insights in temporal progression of DNA-repair and pathway choice. Mutat. Res..

[122] Jakob B., Splinter J., Durante M., Taucher-Scholz G. (2009). Live cell microscopy analysis of radiation-induced DNA double-strand break motion. Proc. Natl. Acad. Sci. USA.

[123] Costes S.V., Ponomarev A., Chen J.L., Nguyen D., Cucinotta F.A., Barcellos-Hoff M.H. (2007). Image-based modeling reveals dynamic redistribution of DNA damage into nuclear sub-domains. PLoS Comput. Biol..

[124] Sundaravinayagam D., Rahjouei A., Andreani M., Tupiņa D., Balasubramanian S., Saha T., Delgado-Benito V., Coralluzzo V., Daumke O., Di Virgilio M. (2019). 53BP1 Supports Immunoglobulin Class Switch Recombination Independently of Its DNA Double-Strand Break End Protection Function. Cell Rep..

[125] Biehs R., Steinlage M., Barton O., Juhász S., Künzel J., Spies J., Shibata A., Jeggo P.A., Löbrich M. (2017). DNA Double-Strand Break Resection Occurs during Non-homologous End Joining in G1 but Is Distinct from Resection during Homologous Recombination. Mol. Cell.

[126] Kallimasioti-Pazi E.M., Chathoth K.T., Taylor G.C., Meynert A., Ballinger T., Kelder M.J.E., Lalevée S., Sanli I., Feil R., Wood A.J. (2018). Heterochromatin delays CRISPR-Cas9 mutagenesis but does not influence the outcome of mutagenic DNA repair. PLoS Biol..

[127] Stadler J., Richly H. (2017). Regulation of DNA Repair Mechanisms: How the Chromatin Environment Regulates the DNA Damage Response. Int. J. Mol. Sci..

[128] Polo S.E., Jackson S.P. (2011). Dynamics of DNA damage response proteins at DNA breaks: A focus on protein modifications. Genes Dev..

[129] Wei L., Lan L., Hong Z., Yasui A., Ishioka C., Chiba N. (2008). Rapid recruitment of BRCA1 to DNA double-strand breaks is dependent on its association with Ku80. Mol. Cell. Biol..

[130] Misteli T., Soutoglou E. (2009). The emerging role of nuclear architecture in DNA repair and genome maintenance. Nat. Rev. Mol. Cell Biol..

[131] Groth A., Rocha W., Verreault A., Almouzni G. (2007). Chromatin Challenges during DNA Replication and Repair. Cell.

[132] Shi L., Oberdoerffer P. (2012). Chromatin dynamics in DNA double-strand break repair. Biochim. Biophys. Acta Gene Regul. Mech..

[133] Klemm S.L., Shipony Z., Greenleaf W.J. (2019). Chromatin accessibility and the regulatory epigenome. Nat. Rev. Genet..

[134] Cremer C., Birk U. (2016). Perspectives in Super-Resolved Fluorescence Microscopy: What Comes Next?. Front. Phys..

[135] Cremer C., Masters B.R. (2013). Resolution enhancement techniques in microscopy. Eur. Phys. J. H.

[136] Kaufmann R., Lemmer P., Gunkel M., Weiland Y., Müller P., Hausmann M., Baddeley D., Amberger R., Cremer C., Enderlein J., Gryczynski Z.K., Erdmann R. (2009). SPDM: Single Molecule Superresolution of Cellular Nanostructures. Single Molecule Spectroscopy and Imaging II, Proceedings of SPIE BiOS, San Jose, CA, USA, 24 February 2009.

[137] Lemmer P., Gunkel M., Weiland Y., Müller P., Baddeley D., Kaufmann R., Urich A., Eipel H., Amberger R., Hausmann M. (2009). Using conventional fluorescent markers for far-field fluorescence localization nanoscopy allows resolution in the 10-nm range. J. Microsc..

[138] Depes D., Lee J.-H., Bobkova E., Jezkova L., Falkova I., Bestvater F., Pagacova E., Kopecna O., Zadneprianetc M., Bacikova A. (2018). Single-molecule localization microscopy as a promising tool for γH2AX/53BP1 foci exploration. Eur. Phys. J..

[139] Hausmann M., Ilić N., Pilarczyk G., Lee J.-H., Logeswaran A., Borroni A.P., Krufczik M., Theda F., Waltrich N., Bestvater F. (2017). Challenges for super-resolution localization microscopy and biomolecular fluorescent nano-probing in cancer research. Int. J. Mol. Sci..

[140] Cremer C., Kaufmann R., Gunkel M., Pres S., Weiland Y., Müller P., Ruckelshausen T., Lemmer P., Geiger F., Degenhard S. (2011). Superresolution imaging of biological nanostructures by spectral precision distance microscopy. Biotechnol. J..

[141] Scherthan H., Lee J.-H., Maus E., Schumann S., Muhtadi R., Chojowski R., Port M., Lassmann M., Bestvater F., Hausmann M. (2019). Nanostructure of Clustered DNA Damage in Leukocytes after In-Solution Irradiation with the Alpha Emitter Ra-223. Cancers.

[142] Natale F., Rapp A., Yu W., Maiser A., Harz H., Scholl A., Grulich S., Anton T., Hörl D., Chen W. (2017). Identification of the elementary structural units of the DNA damage response. Nat. Commun..

[143] Ripley B.D. (1977). Modelling Spatial Patterns. J. R. Stat. Soc. Ser. B.

[144] Hausmann M., Neitzel C., Bobkova E., Nagel D., Hofmann A., Chramko T., Smirnova E., Kopečná O., Pagáčová E., Boreyko A. (2020). Single Molecule Localization Microscopy Analyses of DNA-Repair Foci and Clusters Detected along Particle Damage Tracks. Front. Phys. Sect. Med. Phys. Imaging.

[145] Hofmann A., Krufczik M., Heermann D.W., Hausmann M. (2018). Using Persistent Homology as a New Approach for Super-Resolution Localization Microscopy Data Analysis and Classification of γH2AX Foci/Clusters. Int. J. Mol. Sci..

[146] Iacovoni J.S., Caron P., Lassadi I., Nicolas E., Massip L., Trouche D., Legube G. (2010). High-resolution profiling of γH2AX around DNA double strand breaks in the mammalian genome. EMBO J..

[147] Arnould C., Rocher V., Clouaire T., Caron P., Philippe E.M., Emiliano P.R., Mourad R., Noordermeer D., Legube G. (2020). Loop extrusion as a mechanism for DNA Double-Strand Breaks repair foci formation. bioRxiv.

[148] Zimmermann M., de Lange T. (2014). 53BP1: Prochoice in DNA repair. Trends Cell Biol..

[149] Dutta A., Eckelmann B., Adhikari S., Ahmed K.M., Sengupta S., Pandey A., Hegde P.M., Tsai M.-S., Tainer J.A., Weinfeld M. (2017). Microhomology-mediated end joining is activated in irradiated human cells due to phosphorylation-dependent formation of the XRCC1 repair complex. Nucleic Acids Res..

[150] Ochs F., Karemore G., Miron E., Brown J., Sedlackova H., Rask M.-B., Lampe M., Buckle V., Schermelleh L., Lukas J. (2019). Stabilization of chromatin topology safeguards genome integrity. Nature.

[151] Bártová E., Legartová S., Dundr M., Suchánková J. (2019). A role of the 53BP1 protein in genome protection: Structural and functional characteristics of 53BP1-dependent DNA repair. Aging.

[152] Reindl J., Drexler G.A., Girst S., Greubel C., Siebenwirth C., Drexler S.E., Dollinger G., Friedl A.A. (2015). Nanoscopic exclusion between Rad51 and 53BP1 after ion irradiation in human HeLa cells. Phys. Biol..

[153] Hell S.W., Wichmann J. (1994). Breaking the diffraction resolution limit by stimulated emission: Stimulated-emission-depletion fluorescence microscopy. Opt. Lett..

[154] Varga D., Majoros H., Ujfaludi Z., Erdélyi M., Pankotai T. (2019). Quantification of DNA damage induced repair focus formation via super-resolution dSTORM localization microscopy. Nanoscale.

[155] Wang H., Wang Y. (2014). Heavier Ions with a Different Linear Energy Transfer Spectrum Kill More Cells Due to Similar Interference with the Ku-Dependent DNA Repair Pathway. Radiat. Res..

[156] Xie M., Park D., You S., Li R., Owonikoko T.K., Wang Y., Doetsch P.W., Deng X. (2015). Bcl2 inhibits recruitment of Mre11 complex to DNA double-strand breaks in response to high-linear energy transfer radiation. Nucleic Acids Res..

[157] Roobol S.J., van den Bent I., van Cappellen W.A., Abraham T.E., Paul M.W., Kanaar R., Houtsmuller A.B., van Gent D.C., Essers J. (2020). Comparison of High- and Low-LET Radiation-Induced DNA Double-Strand Break Processing in Living Cells. Int. J. Mol. Sci..

[158] Scuric Z., Chan C.Y., Hafer K., Schiestl R.H. (2009). Ionizing Radiation Induces Microhomology-Mediated End Joining *in trans* in Yeast and Mammalian Cells. Radiat. Res..

[159] Shibata A., Conrad S., Birraux J., Geuting V., Barton O., Ismail A., Kakarougkas A., Meek K., Taucher-Scholz G., Löbrich M. (2011). Factors determining DNA double-strand break repair pathway choice in G2 phase: DSB repair pathway choice in G2 phase. EMBO J..

[160] Jeggo P.A., Löbrich M. (2017). DNA non-homologous end-joining enters the resection arena. Oncotarget.

[161] Guckenberger M., Combs S.E., Zips D. (2018). Advances in Radiation Therapy.

[162] Lorat Y., Brunner C.U., Schanz S., Jakob B., Taucher-Scholz G., Rübe C.E. (2015). Nanoscale analysis of clustered DNA damage after high-LET irradiation by quantitative electron microscopy–The heavy burden to repair. DNA Repair.

[163] Tang N., Bueno M., Meylan S., Incerti S., Tran H.N., Vaurijoux A., Gruel G., Villagrasa C. (2019). Influence of chromatin compaction on simulated early radiation-induced DNA damage using Geant4-DNA. Med. Phys..

[164] Shibata A., Jeggo P.A. (2020). Canonical DNA non-homologous end-joining; capacity versus fidelity. Br. J. Radiol..

[165] Iliakis G. (2018). The Biological Foundations of Risks from Ionizing Radiation Exposures: How an Understanding of Associated Effects Will Help Their Quantification and Mitigation. Sustainable Risk Management.

[166] Cremer T., Cremer M., Hübner B., Silahtaroglu A., Hendzel M., Lanctôt C., Strickfaden H., Cremer C. (2020). The Interchromatin Compartment Participates in the Structural and Functional Organization of the Cell Nucleus. BioEssays.

[167] Bendandi A., Dante S., Zia S.R., Diaspro A., Rocchia W. (2020). Chromatin Compaction Multiscale Modeling: A Complex Synergy Between Theory, Simulation, and Experiment. Front. Mol. Biosci..

[168] Van Steensel B., Furlong E.E.M. (2019). The role of transcription in shaping the spatial organization of the genome. Nat. Rev. Mol. Cell Biol..

[169] Vergara Z., Gutierrez C. (2017). Emerging roles of chromatin in the maintenance of genome organization and function in plants. Genome Biol..

[170] Hauer M.H., Gasser S.M. (2017). Chromatin and nucleosome dynamics in DNA damage and repair. Genes Dev..

[171] Zafar F., Okita A.K., Onaka A.T., Su J., Katahira Y., Nakayama J., Takahashi T.S., Masukata H., Nakagawa T. (2017). Regulation of mitotic recombination between DNA repeats in centromeres. Nucleic Acids Res..

[172] Janssen A., Colmenares S.U., Karpen G.H. (2018). Heterochromatin: Guardian of the Genome. Annu. Rev. Cell Dev. Biol..

[173] Allshire R.C., Madhani H.D. (2018). Ten principles of heterochromatin formation and function. Nat. Rev. Mol. Cell Biol..

[174] Jasin M., Rothstein R. (2013). Repair of strand breaks by homologous recombination. Cold Spring Harb. Perspect. Biol..

[175] Pagáčová E., Falk M., Falková I., Lukášová E., Michalová K., Oltová A., Raška I., Kozubek S. (2014). Frequent chromatin rearrangements in myelodysplastic syndromes–what stands behind?. Folia Biol..

[176] Koltsova A.S., Pendina A.A., Efimova O.A., Chiryaeva O.G., Kuznetzova T.V., Baranov V.S. (2019). On the Complexity of Mechanisms and Consequences of Chromothripsis: An Update. Front. Genet..

[177] Lin F.L., Sperle K., Sternberg N. (1984). Model for homologous recombination during transfer of DNA into mouse L cells: Role for DNA ends in the recombination process. Mol. Cell. Biol..

[178] Cornforth M.N., Durante M. (2018). Radiation quality and intra-chromosomal aberrations: Size matters. Mutat. Res. Genet. Toxicol. Environ. Mutagenesis.

[179] Brenner D.J., Okladnikova N., Hande P., Burak L., Geard C.R., Azizova T. (2001). Biomarkers Specific to Densely-ionising (High LET) Radiations. Radiat. Prot. Dosim..

[180] Costes S.V., Chiolo I., Pluth J.M., Barcellos-Hoff M.H., Jakob B. (2010). Spatiotemporal characterization of ionizing radiation induced DNA damage foci and their relation to chromatin organization. Mutat. Res. Rev. Mutat. Res..

[181] Banno K., Kisu I., Yanokura M., Tsuji K., Masuda K., Ueki A., Kobayashi Y., Yamagami W., Nomura H., Tominaga E. (2012). Epimutation and cancer: A new carcinogenic mechanism of Lynch syndrome. Int. J. Oncol..

[182] Machnik M., Oleksiewicz U. (2020). Dynamic Signatures of the Epigenome: Friend or Foe?. Cells.

[183] Lønning P.E., Eikesdal H.P., Løes I.M., Knappskog S. (2019). Constitutional Mosaic Epimutations–a hidden cause of cancer?. Cell Stress.

[184] Misteli T. (2010). Higher-order genome organization in human disease. Cold Spring Harb. Perspect. Biol..

[185] Engreitz J.M., Agarwala V., Mirny L.A. (2012). Three-Dimensional Genome Architecture Influences Partner Selection for Chromosomal Translocations in Human Disease. PLoS ONE.

[186] Iarovaia O.V., Rubtsov M., Ioudinkova E., Tsfasman T., Razin S.V., Vassetzky Y.S. (2014). Dynamics of double strand breaks and chromosomal translocations. Mol. Cancer.

[187] Chagin V.O., Reinhart B., Becker A., Mortusewicz O., Jost K.L., Rapp A., Leonhardt H., Cardoso M.C. (2019). Processive DNA synthesis is associated with localized decompaction of constitutive heterochromatin at the sites of DNA replication and repair. Nucleus.

[188] Bach M., Savini C., Krufczik M., Cremer C., Rösl F., Hausmann M. (2017). Super-Resolution Localization Microscopy of γ-H2AX and Heterochromatin after Folate Deficiency. Int. J. Mol. Sci..

[189] Cann K.L., Dellaire G. (2011). Heterochromatin and the DNA damage response: The need to relax. Biochem. Cell Biol..

[190] Nickoloff J.A., De Haro L.P., Wray J., Hromas R. (2008). Mechanisms of leukemia translocations. Curr. Opin. Hematol..

[191] Kozubek S., Bártová E., Kozubek M., Lukásová E., Cafourková A., Koutná I., Skalníková M. (2001). Spatial distribution of selected genetic loci in nuclei of human leukemia cells after irradiation. Radiat. Res..

[192] Kozubek S., Lukásová E., Marecková A., Skalníková M., Kozubek M., Bártová E., Kroha V., Krahulcová E., Slotová J. (1999). The topological organization of chromosomes 9 and 22 in cell nuclei has a determinative role in the induction of t(9,22) translocations and in the pathogenesis of t(9,22) leukemias. Chromosoma.

[193] Lukášová E., Kozubek S., Kozubek M., Kroha V., Marečková A., Skalníková M., Bártová E., Šlotová J. (1999). Chromosomes participating in translocations typical of malignant hemoblastoses are also involved in exchange aberrations induced by fast neutrons. Radiat. Res..

[194] Nathanailidou P., Taraviras S., Lygerou Z. (2020). Chromatin and Nuclear Architecture: Shaping DNA Replication in 3D. Trends Genet..

[195] Meaburn K.J., Misteli T., Soutoglou E. (2007). Spatial genome organization in the formation of chromosomal translocations. Semin. Cancer Biol..

[196] Rowley M.J., Corces V.G. (2018). Organizational principles of 3D genome architecture. Nat. Rev. Genet..

[197] Schmitt A.D., Hu M., Ren B. (2016). Genome-wide mapping and analysis of chromosome architecture. Nat. Rev. Mol. Cell Biol..

[198] Takata H., Hanafusa T., Mori T., Shimura M., Iida Y., Ishikawa K., Yoshikawa K., Yoshikawa Y., Maeshima K. (2013). Chromatin Compaction Protects Genomic DNA from Radiation Damage. PLoS ONE.

[199] Krigerts J., Salmina K., Freivalds T., Rumnieks F., Inashkina I., Zayakin P., Hausmann M., Erenpreisa J. (2020). Early Critical Phase Transitions of Pericentromere-Associated Domains in MCF-7 Breast Cancer Cells Committed to Differentiation by Heregulin. Preprints.

[200] Williamson A.K., Zhu Z., Yuan Z.-M. (2018). Epigenetic mechanisms behind cellular sensitivity to DNA damage. CST.

[201] Matsuoka S., Ballif B.A., Smogorzewska A., McDonald E.R., Hurov K.E., Luo J., Bakalarski C.E., Zhao Z., Solimini N., Lerenthal Y. (2007). ATM and ATR Substrate Analysis Reveals Extensive Protein Networks Responsive to DNA Damage. Science.

[202] Hildenbrand G., Metzler P., Pilarczyk G., Bobu V., Kriz W., Hosser H., Fleckenstein J., Krufczik M., Bestvater F., Wenz F. (2018). Dose enhancement effects of gold nanoparticles specifically targeting RNA in breast cancer cells. PLoS ONE.

